# Availability of Antibiotics for Veterinary Use on the Internet: A Cross-Sectional Study

**DOI:** 10.3389/fvets.2021.798850

**Published:** 2022-02-11

**Authors:** Juan F. Garcia, M. Jose Diez, Ana M. Sahagun, Raquel Diez, Matilde Sierra, Juan J. Garcia, Cristina López, M. Nelida Fernandez

**Affiliations:** ^1^Department of Mechanical, Informatics and Aerospatiale Engineering, University of Leon, Leon, Spain; ^2^Department of Biomedical Sciences, Institute of Biomedicine (IBIOMED), Veterinary Faculty, University of Leon, Leon, Spain

**Keywords:** antimicrobials, one health, antibiotics, veterinary, resistance

## Abstract

The misuse or overuse of antibiotics can favor the emergence of antimicrobial resistance, with a direct impact on human and animal health as well as on ecosystems. In this work, we have analyzed the web pages in which antibiotics for veterinary use can be purchased online. By using a specifically developed software, we carried out a detailed search to know if each individual antibiotic and the families included in the OIE list of antimicrobial agents of veterinary importance (in English and in Spanish) were sold, reviewing the offers according to the administration route and the species for which the antibiotics were intended. The greatest offer of antibiotics was for those compounds considered critically important by OIE. In the search in English, penicillins were available on 55.8% of the sites, tetracyclines on 55.0%, and fluoroquinolones on 52.7%. In Spanish, the families with the greatest presence were fluoroquinolones (67.9% of the sites), tetracyclines (67.9% of the sites), and penicillins (65.4% of the sites). Regarding individual antibiotics, the most offered in both searches were amoxicillin (49.6% of the sites in English and 57.7% in Spanish) and doxycycline (46.5% of the sites in English and 53.8% in Spanish). Most offers were for oral and parenteral administration and intended for cats and dogs and for bovines.

## Introduction

Online purchasing of prescription medications has grown significantly and has been intensified by social isolation related to the COVID-19 pandemic (1). Several studies have demonstrated that prescription drugs can be purchased online, like stimulants, opioids, sedatives, or antibiotics (2–7). Buying medicines online have got undoubtful advantages for consumers: purchasing at anytime and anywhere, privacy, convenience, the possibility of comparing prices or having easy access to a broader range of products, sharing opinions, free access to information, no barrier of distance, discounts, etc. However, they also bring inconveniences: Beyond the possibility of acquiring counterfeit medicines or abuse drugs, the potential absence of diagnosis or professional supervision of the treatments and the access to incorrect or inappropriate information on medicines (sometimes websites retain information on drug adverse effects, contraindications, or interactions) should be also kept in mind (8–12).

Antibiotics are among those drugs that can be accessed online, both in legal and illegal websites. These medicines have been of great benefit against human and animal infectious diseases, and they have saved a large number of lives. Nevertheless, it is acknowledged that their misuse or overuse in different sectors (animal, human, and agriculture) has favored or accelerated the emergence of antimicrobial resistance (13), with a direct impact on human and animal health as well as on ecosystems (14). Antimicrobial resistance is one of the main issues included in “One Health” concept (15), having become one of the priorities for international and national authorities.

Online marketing has been identified as one of the factors which promote antimicrobial resistance, by making restricted antibiotics accessible (16–19). The World Health Organization (WHO) has drawn attention to the need to control the use of antimicrobial medicines in both human and animal health, the inadequate or poor regulation on their online sales, and the ease of buying them over the Internet without prescription from or the involvement of a doctor/veterinarian (20). Despite the fact that international organizations and countries have established targets to reduce the use of antibiotics, few control measures for the sale of antibiotics on the Internet have been implemented, thus limiting the impact of the interventions developed (21). We have carried out a previous study (2) in which we evaluated the availability of the 5 families classified as highest priority critically important antimicrobials. However, antibiotic resistance is established by individual compounds, not by family; so, to better establish the magnitude of the problem, it is important to know the offer of the individual compounds.

In this sense, the aims of this study were as follows: (1) to study the sale characteristics of the web pages where antibiotics can be purchased and (2) to evaluate the number of web pages that offered each family and individual antibiotic included in the OIE list of antimicrobial agents of veterinary importance (18), their availability for different administration routes, and the species for which they were intended.

To perform the search, a specifically designed software was developed.

## Materials and Methods

This study was carried out by a multidisciplinary working group that included both pharmacology experts (pharmacology professors from the Veterinary Faculty at the University of León, Spain: MS, JJG, MJD, AS, RD, and MNF) and a member with expertise in cybersecurity (School of Industrial, Informatics and Aerospatiale Engineering: JFG). JFG developed the search software and programmed the searches. The pharmacology experts reviewed and evaluated, by pairs, the data obtained. Any potential disagreement was resolved by all members. The searches and the data evaluation took 10 days.

### Design

This cross-sectional study was conducted as being an observation at a point in time of the online availability of the families and each individual antibiotic included in the OIE list of antimicrobial agents of veterinary importance (22). The Strengthening the Reporting of Observational Studies in Epidemiology statement was used to report data (23), and the checklist was included in the Supplementary Material.

### Data Obtained From the Website Pages Analyzed

We evaluated the number of web pages that offered each family and compound included in the OIE list of antimicrobial agents of veterinary importance (22). The website pages selected were also examined to determine the availability of antibiotics for different administration routes—oral, parenteral, topical, intramammary, intrauterine, or not specified—and the species for which the antibiotics were intended. The categories of species were established considering the sale specifications of the web pages and were as follows: bovine, ovine, caprine, pigs, horses, poultry, dogs, fish, and humans, when the products were sold only for the species mentioned; cats and dogs, animals, and humans, when the formulations could be used for both; all species, when the web page indicated that the product could be used in all species; and others, for other species like rabbits or bees. The “humans” category has been included because, although they have this specific characteristic of sale in the web pages, those antibiotics can also be used in animals, especially in pet dogs and cats.

Before examining the offer of antibiotics, the characteristics of the websites found were assessed: if the web page connected or not, whether or not they sold antibiotics, and in those in which antibiotics could be purchased, the requirement of prescription, if it was for veterinary professionals only or if it was not explained how they sold the antibiotics, and the country from which webs sell and the countries the vendor would sell antibiotics to.

### Search Engine

The automated searches were carried out by using a specifically designed software created by the cybersecurity expert (Mirkwood crawler). This software simplified the finding, extraction, and analysis of a high amount of relevant information available in online websites, no matter how deep the links within the sites were. Web harvesting, scratching, or harvesting alludes to information extraction from websites (24); it can be done either manually or using a specific software bot, usually called a web crawler. This type of software can visit the whole website while optionally downloading each of its pages to a local drive for later analysis (this type of analysis is referred to as offline analysis). For our study, we designed and implemented an advanced crawler which can run in parallel (taking advantage of clusters of computers in the same local network as well as using all the cores and processors of each individual machine) and analyze sites “on the fly,” that is, online and dynamically at the very same time that it visits each of the pages of the sites without downloading them. Our software can also set up the list of websites to visit (called “seeds”) all by itself only by providing it with a simple or complex search query. All these advanced features directly translate into a great speeding up of the search and analysis process while guaranteeing that we always work with updated content. We called our harvester “Mirkwood” as a tribute to Lord of the Rings and The Hobbit books: it is the title of a woodland of middle-earth whose pockets were ruled by creepy crawlies (25). The name suits our tool since its architecture is based upon spiders, their nests, and a forest which include them all (for a more detailed explanation of Mirkwood and its underlying technology, please refer to the Supplementary Material).

Our crawler can analyze sites searching for simple patterns, but we still rely on expert validation for studies that are more complex as well as for validation and correct interpretation of the results of the crawler. As we have indicated before, we included as search terms the whole list of the antibiotics included in the OIE list of antimicrobial agents of veterinary importance (22), which are listed in Table 1, both in English and Spanish.

**Table 1:** Families of antibiotics and individual compounds used as search terms.

**Family**	**Individual compounds**
**Critically important**	
Aminoglycosides	Aminocyclitol, amikacin, apramycin, dihydrostreptomycin, fortimycin, framycetin, gentamicin, kanamycin, neomycin, paromomycin, spectinomycin, streptomycin, tobramycin
Amphenicols	Florphenicol, thiamphenicol
Cephalosporins	Cefoperazone, ceftiofur, ceftriaxone, cefquinome
Macrolides	Carbomycin, erythromycin, gamithromycin, josamycin, kitasamycin, mirosamycin, oleandomycin, sedecamycin, spiramycin, terdecamycin, tildipirosin, tilmicosin, tulathromycin, tylosin, tylvalosin
Penicillins	Natural penicillins: benethamine penicillin, benzylpenicillin, penethamate, benzylpenicillin procaine, benzathine penicillin; amdinopenicillins: mecillinam; aminopenicillins: amoxicillin, amoxicillin/clavulanic acid, ampicillin, ampicillin/sulbactam, hetacillin; carboxypenicillins: ticarcillin, tobicillin; ureidopenicillin: aspoxicillin; phenoxypenicillins: phenoxymethylpenicillin, phenethicillin; antistaphylococcal penicillins: cloxacillin, dicloxacillin, nafcillin, oxacillin
Fluoroquinolones	Ciprofloxacin, danofloxacin, difloxacin, enrofloxacin, marbofloxacin, norfloxacin, ofloxacin, orbifloxacin, sarafloxacin
Sulfonamides	Phthalylsulfathiazole, sulfachlorpyridazine, sulfadiazine, sulfadimerazin, sulfadimethoxazole, sulfadimethoxine, sulfadimidine, sulfadoxine, sulfafurazole, sulfaguanidine, sulfamerazine, sulfamethazine, sulfamethoxine, sulfamethoxypyridazine, sulfamonomethoxine, sulfanilamide, sulfapyridine, sulfaquinoxaline
Diaminopyrimidines	Baquiloprim, trimethoprim, ormetoprim
Sulfonamides + diaminopyrimidines	Sulfadimethoxine/ormetoprim, sulfonamide/trimethoprim
Tetracyclines	Chlortetracycline, doxycycline, oxytetracycline, tetracycline
**Highly important**	
Ansamycin–rifamycins	Ansamycin, rifampicin, rifaximin
Cephalosporins	Cefacetrile, cefalexin, cefalotin, cefapyrin, cefazolin, cefalonium, cefuroxime
Ionophores	Lasalocid, maduramycin, monensin, narasin, salinomycin, semduramicin
Lincosamides	Pirlimycin, lincomycin
Phosphonic acid derivates	Phosphonic acid, fosfomycin
Pleuromutilins	Tiamulin, valnemulin
Polypeptides	Enramycin, gramicidin, bacitracin, colistin, polymixin
Quinolones	Flumequin, miloxacin, nalidixic acid, oxolinic acid
**Important**	
Aminocoumarin	Aminocoumarin, novobiocin
Arsenical	Roxarsone, nitarsone
Bicyclomycin	Bicyclomycin, bicozamycin
Fusidic acid	Fusidic acid
Orthosomycins	Avilamycin
Quinoxalines	Carbadox, olaquindox
Streptogramins	Virginiamycin
Thiostrepton	Thiostrepton, nosiheptide

*Adapted from the OIE list of antimicrobial agents of veterinary importance (22)*.

Regarding specific technologies, we programmed our software using Java Standard Edition, version 8. Since Java is an object-oriented language, each module is implemented as a class. Our software is portable and can work on a large number of platforms and operating systems given the nature of Java. While the crawler can use all the power of computer clusters, it can also optimally run in isolated machines by means of threading. We use java.lang.Runnable for Thread implementation (allowing communication between all the spiders of a nest). Interlocking and race conditions are prevented by semaphores. MPI [we specifically use Open MPI (26)] allows for communication between machines in the cluster (between the nests and the forest). To visit the websites and extract their page contents, we have to deal with HTML, and we do so using Jsoup, a library that supports different versions of HTML and creates parse trees (27, 28). Our crawler can work as a general purpose harvester or as a focused one, that is, a crawler that does not try to visit the whole Internet but just a reduced set of domains.

The program (Mirkwood crawler) in a ready-to-use state is available upon request to the author (JFG) only for research purposes (profit- or commercial-driven use of the software is strictly forbidden).

## Results

A total of 261 websites were analyzed in detail; 160 of them were obtained after the search was carried out in English and 101 in Spanish.

### Web Page Characteristics

The characteristics of each web page were reviewed, and the results obtained are included in Table 2. After the search carried out in English, we found that 19 pages did not connect (11.9%), and 12 of the websites (7.5%) did not sell antibiotics. The Spanish search results were similar; 15 pages did not connect (14.9%), and 8 did not sell antibiotics (7.9%). Regarding the web pages that did not sell antibiotics, those in English operated from the USA (6 pages), Canada (3 pages), United Kingdom (1 page), China (1 page), and India (1 page) and those in Spanish were 3 from Mexico, 2 from Spain, 1 from Peru, and 1 from Argentina. So, 80.6% of the web pages reviewed in English and 77.2% of those in Spanish sold antibiotics.

**Table 2:** Changes in the requirement of prescription to purchase the antibiotics of the web pages analyzed in 2020 (2) and in 2021.

			**Year 2021**							
			**Prescription required**		**Prescription not required**		**Mixed**		**Not explained**	
			**E: *n* = 37**	**S: *n* = 18**	**E: *n* = 76**	**S: *n* = 48**	**E: *n* = 2**	**S: *n* = 1**	**E: *n* = 7**	**S: *n* = 6**
Year 2020	Prescription required	E: *n* = 33	27		4		1		1	
		S: *n* = 15		12		0		0		3
	Prescription not required	E: *n* = 75	5		67		1		2	
		S: *n* = 51		3		45		0		3
	Mixed	E: *n* = 4	2		1		1		0	
		S: *n* = 3		1		1		1		0
	Not explained	E: *n* = 9	3		4		0		2	
		S: *n* = 4		2		2		0		0

*Shaded cells indicate no changes, whereas unshaded cells indicate changes*.

*E, search in English; S, search in Spanish; Mixed, prescription required for some antibiotics and prescription not required for others*.

Tables 3, 4 include the comparison of the web pages analyzed in 2020, in our previous study (5), and in 2021. Few changes in the requirement of prescription were found (Table 3). Five of the web pages in English and three of those in Spanish required a prescription, while in 2020 they did not, and another three in English and two in Spanish asked the prescription, while in 2020 they did not explain it.

**Table 3:** Changes in the countries from which web pages sold and the countries they sold to of the web pages analyzed in 2020 (2) and in 2021.

	**Search in English**	**Search in Spanish**
Change in the country they sold from	22	8
Change in the countries they sold to	17	10
More countries they sell to	4	1
Less countries they sell to	13	9

**Table 4:** Characteristics of the web pages reviewed.

	**Search in English** **(*n* = 160)**	**Search in Spanish** **(*n* = 101)**
Do not connect	19	15
Do not sell antibiotics	12	8
Sell antibiotics	129	78
• Prescription required	37	18
• No prescription required	76	48
• With and without prescription	2	1
• For veterinary professionals	7	5
• Not explained	7	6

Regarding the countries which the websites sold from and those they sell to (Table 4), 22 sites in English and 8 in Spanish changed the country they sold from, and all of them did not require a prescription. Most sites in English changed from Canada to USA or from USA to Canada. In relation to the changes in the number of countries they sold to (17 sites), most of them were sites that restricted the number of countries they sold to: 13 webs in English and 9 in Spanish.

### Antibiotic Availability

Table 5 includes the number of websites where antibiotics can be purchased according to the family to which they belong following the OIE list of antimicrobial agents of veterinary importance (25), in the searches in English and in Spanish. In both searches, although in different order, the most frequently sold families were penicillins, tetracyclines, fluoroquinolones, and aminoglycosides (all of them are considered critically important), with very similar percentages in the offer. Regarding individual antibiotics (Supplementary Table S1), amoxicillin and doxycycline were the most commonly offered in both searches, also belonging to the category of critically important antibiotics.

**Table 5:** Number of websites where the families of antibiotics can be purchased, classified following the OIE list of antimicrobial agents of veterinary importance (22), in the searches in English and in Spanish.

	**Search in English (*n* = 129)**	**Search in Spanish (*n* = 78)**	**Total (*n* = 207)**
**Critically important**			
Aminoglycosides	37	45	82
Amphenicols	5	16	21
Cephalosporins	11	20	31
Macrolides	57	40	97
Penicillins	72	51	123
Fluoroquinolones	68	53	121
Sulfonamides	35	41	76
Diaminopyrimidines	9	6	15
Sulfonamides + diaminopyrimidines	50	26	76
Tetracyclines	71	53	124
**Highly important**			
Ansamycin–rifamycins	14	7	21
Cephalosporins	56	36	92
Ionophores		1	1
Lincosamides	29	19	48
Phosphonic acid	1	3	4
Pleuromutilins	3	4	7
Polypeptides	23	19	42
Quinolones	6	3	9
**Important**			
Aminocoumarin			
Arsenical			
Bicyclomycin			
Fusidic acid	6	2	8
Orthosomycins			
Quinoxalines			
Streptogramins			
Thiostrepton	2	2	4

*The names of the individual compounds included in each family are detailed in Table 1. The number of websites where each individual antibiotic can be purchased are included in Supplementary Table S1*.

*Cells shaded in gray indicate zero offers*.

The sale characteristics of the different families of antibiotics are shown in Table 6 (routes of administration for which the antibiotics are available) and Table 7 (species to which they are intended for). After the searches carried out in English and in Spanish, we found that the most frequent formulations available were for oral or parenteral administration. Regarding the species they were intended for, formulations for humans were the most usual in English, followed by those for cats and dogs and for bovine and in Spanish were for cats and dogs, followed by those for bovine and pigs.

**Table 6:** Distribution of antibiotic offers, according to the administration route and classified following the OIE list of antimicrobial agents of veterinary importance (22), in the searches in English and in Spanish.

	**Oral**			**Parenteral**			**Topical**			**Intramammary**			**Intrauterine**			**Unknown**		
	**E**	**S**	**T**	**E**	**S**	**T**	**E**	**S**	**T**	**E**	**S**	**T**	**E**	**S**	**T**	**E**	**S**	**T**
**Critically important**																		
Aminoglycosides	27	18	45	20	68	88	36	61	97	2	21	23	1	4	5	7		7
Amphenicols		9	9	4	11	15		1	1							1		1
Cephalosporins	3		3	9	23	32		1	1	2	2	4						
Macrolides	55	40	95	22	25	47	3	5	8		9	9				3		3
Penicillins	165	83	248	36	85	121	1	6	7	5	25	30		2	2	2		2
Fluoroquinolones	128	80	208	13	23	36	24	31	55		1	1				7		7
Sulfonamides	31	68	99	10	22	32	6	15	21		1	1				3		3
Diaminopyrimidines	7	4	11	2	2	4												
Sulfonamides + diaminopyrimidines	49	24	73	3	7	10	1	1	2		1	1				2		2
Tetracyclines	116	69	185	16	25	41	10	13	23		1	1		2	2	3		3
**Highly important**																		
Ansamycin–rifamycins	18	3	21					2	2		2	2		2	2			
Cephalosporins	76	37	113	3	9	12		1	1	2	6	8		2	2			
Ionophores	0	1	1	0	0	0												
Lincosamides	22	13	35	8	8	16	1		1	1	2	3				1		1
Phosphonic acid	1	3	4															
Pleuromutilins	1	4	5	1	2	3										2		2
Polypeptides	5	3	8	3	4	7	33	26	59	0	1	1				3		3
Quinolones	7	3	10															
**Important**																		
Aminocoumarin																		
Arsenical																		
Bicyclomycin																		
Fusidic acid	1	1	2				6	1	7									
Orthosomycins																		
Quinoxalines																		
Streptogramins																		
Thiostrepton							2	2	4									

*The numerical value corresponds to the sum of the web pages where each of the individual antibiotics included in the family is sold. The names of the individual compounds included in each family are detailed in Table 1. The number of websites selling each individual antibiotic according to the administration route are included in Supplementary Table S2*.

*Cells shaded in gray indicate zero offers*.

*E, search in English; S, search in Spanish; T, total*.

**Table 7:** Distribution of the antibiotic offers, according to the species they are intended to and classified following the OIE list of antimicrobial agents of veterinary importance (22), in the searches in English and in Spanish.

	**All species**			**Bovine**			**Ovine**			**Caprine**			**Pigs**			**Horses**			**Poultry**			**Dogs**			**Cats and dogs**			**Fish**			**Humans**			**Animals and humans**			**Others**		
	**E**	**S**	**T**	**E**	**S**	**T**	**E**	**S**	**T**	**E**	**S**	**T**	**E**	**S**	**T**	**E**	**S**	**T**	**E**	**S**	**T**	**E**	**S**	**T**	**E**	**S**	**T**	**E**	**S**	**T**	**E**	**S**	**T**	**E**	**S**	**T**	**E**	**S**	**T**
**Critically important**																																							
Aminoglycosides	6	25	31	11	54	65	6	34	40	8	30	38	10	37	47	14	38	52	8	14	22	6	12	18	25	56	81				26	13	39	7	2	9	11	7	18
Amphenicols	1	2	3	4	12	16		2	2					12	12		1	1		4	4		1	1	2	1	3											2	2
Cephalosporins	1	2	3	6	18	24	1		1	1		1	2	17	19	3	5	8	1	5	6		1	1	1	2	3				5	3	8						
Macrolides		7	7	16	31	47	2	7	9	1	4	5	13	16	29	2		2	2	11	13	1	2	3	12	23	35	6		6	35	8	43	3		3	6	4	10
Penicillins	3	22	25	20	73	93	10	41	51	3	26	29	22	43	65	12	32	44	1	10	11	4	3	7	24	69	93	24		24	127	39	166	2	3	5	5	1	6
Fluoroquinolones	2	7	9	9	19	28	1	10	11		6	6	5	16	21	1	8	9	1	18	19		10	10	32	58	90	12		12	84	26	110	3	1	4	3	5	8
Sulfonamides	3	10	13	12	33	45	4	22	26	2	17	19	1	26	27	5	27	32	5	13	18	1	17	18	11	30	41	1	2	3	5	8	13	2	4	6	5	4	9
Diaminopyrimidines					4	4										2	5	7							3	7	10	2		2	12	1	13						
Sulfonamides + diaminopyrimidines	1	4	5	1	9	10	2	9	11		5	5		11	11	4	8	12		6	6				6	9	15	7		7	21	5	26	1		1	5	5	10
Tetracyclines	3	8	11	21	32	53	7	24	31	4	16	20	16	28	44	5	17	22	13	21	34	3		3	17	41	58	11		11	60	18	78	2	1	3	19	10	29
**Highly important**																																							
Ansamycin–rifamycins					3	3										1	2	3								1	1	1		1	16	3	19						
Cephalosporins		1	1	2	11	13	1	2	3		1	1		2	2	2	4	6				1		1	13	20	33	15		15	64	19	83						
Ionophores					1	1																																	
Lincosamides		1	1	1	8	9		3	3		2	2	7	8	15	1	2	3	5	6	11		1	1	2	8	10				14	6	20				3		3
Phosphonic acid					1	1		1	1					3	3					3	3					1	1				1		1					3	3
Pleuromutilins				1		1							2	4	6					4	4																		
Polypeptides	2	4	6	3	7	10	3	3	6				1	4	5	4	2	6	1	4	5		1	1	18	21	39				15	4	19	2		2		1	1
Quinolones																															7	3	10						
**Important**																																							
Aminocoumarin																																							
Arsenical																																							
Bicyclomycin																																							
Fusidic acid																															6	2	8						
Orthosomycins																																							
Quinoxalines																																							
Streptogramins																																							
Thiostrepton	1		1																						1	2	3												

*The numerical value corresponds to the sum of the web pages where each of the individual antibiotics included in the family is sold. The names of the individual compounds included in each family are detailed in Table 1. The number of websites selling each individual antibiotic according to the species they are intended to are included in Supplementary Table S3*.

*Cells shaded in gray indicate zero offers*.

*E, search in English; S, search in Spanish; T, total*.

The results obtained for the different families and individual antibiotics included in each category (critical importance, highly important, and important) are analyzed below.

### Critically Important Antibiotics

In the search in English, the most frequently offered families of this group (Table 3) were penicillins (55.8% of the sites) and tetracyclines (55.0% of the sites). In Spanish, the families with the greatest presence belonged to this group and were fluoroquinolones (67.9% of the sites) and tetracyclines (67.9% of the sites). Considering the antibiotics individually (Supplementary Table S1), the most frequently offered in both searches were amoxicillin (49.6 and 57.7% of the sites in English and in Spanish, respectively) and doxycycline (46.5 and 53.8% of the sites in English and in Spanish, respectively).

Taking into account the route of administration (Table 6), we could see that the most commonly offered formulations were for oral administration, followed by those for the parenteral and topical routes. Considering the different antibiotic families, after the search in English, we found that the most commonly offered formulations were penicillins (165 offers) and fluoroquinolones (128 offers) for oral administration. In Spanish, the most frequent were penicillins for parenteral and oral administration (85 and 83 offers, respectively). Regarding individual compounds (Supplementary Table S2), they were amoxicillin (48.1 and 50.0% of the sites in English and in Spanish, respectively) and doxycycline (45.0 and 46.2% of the sites in English and in Spanish, respectively) for oral administration.

Regarding the species they were intended for (Table 7), most formulations were intended for humans in English, followed by those for cats and dogs, and for cats and dogs in Spanish, followed by those for bovine. If the sites intended for humans are not considered, the greatest offer in English is also for cats and dogs. Many of the websites in English were pages that sold drugs for humans in single pills; so as to avoid the influence of these sites in the results, the data obtained without “humans” is also explained. The most usual formulations in English considering the families were penicillins (127 offers) and fluoroquinolones (84 offers), all of them for humans, and the individual antibiotics amoxicillin (28.7% of the sites) and ciprofloxacin (27.1% of the sites), also for humans. Eliminating the category “humans,” the order for families was as follows: fluoroquinolones (35 offers) and aminoglycosides for cats and dogs (25 offers); for individual antibiotics: oxytetracycline for bovine (11.6% of the sites) and neomycin for cats and dogs (10.9% of the sites). In Spanish, the most commonly offered families were penicillins for bovine (73 offers) and penicillins and fluoroquinolones for cats and dogs (69 and 58 offers, respectively); for the individual antibiotics (Supplementary Table S3), these were amoxicillin (32.1% of the sites) and gentamicin and neomycin (24.3% of the sites) for cats and dogs.

### Highly Important Antibiotics

In the category of highly important antibiotics, in both searches, the most usually available families (Table 7) were cephalosporins (43.4% of the sites in English and 46.2% of the sites in Spanish) and lincosamides (22.5% of the sites in English and 24.4% of the sites in Spanish). The individual antibiotics offered in the largest number of webs (Supplementary Table S1) were cefalexin (39.5 and 41.0% of the sites in English and in Spanish, respectively) and lincomycin (21.7 and 24.4% of the sites in English and in Spanish, respectively). Only 1 web offered an antibiotic belonging to the ionophores family (salynomicin), and it was found in the search carried out in Spanish.

As in the category of critically important antibiotics, the most commonly offered formulations were for oral administration. In relation to the families evaluated, oral cephalosporins (76 and 37 offers in English and in Spanish, respectively) and oral lincosamides (22 and 13 offers in English and in Spanish, respectively) were the most usually offered dosage forms (Table 6). The results for the individual antibiotics (Supplementary Table S2) were oral cefalexin (39.5 and 34.6% of the sites in English and in Spanish, respectively), lincomycin (17.1 and 16.7% of the sites in English and in Spanish, respectively).

Taking into account the species which the formulations are intended for (Table 7), most offers in English were for humans and in Spanish for cats and dogs. If “humans” are not considered, cats and dogs are also the most obtainable in English. Considering the families of antibiotics, after the search in English, we found that cephalosporins for humans (64 offers) and polypeptides for cats and dogs (18 offers) were the most commonly available in the websites, and for the individual antibiotics, these were as follows: cefuroxime (24.0% of the sites) and cefalexine (23.3% of the sites) for humans. If we do not consider “humans,” the most commonly offered are polymixin for cats and dogs (9.3% of the sites) and cephalexin for fish (9.3% of the sites). After the search in Spanish, the results for the families were as follows: polypeptides and cephalosporins for cats and dogs (21 and 20 offers, respectively) and cephalosporins for humans (19 offers); for the individual antibiotics (Supplementary Table S3), these were cephalexin for cats and dogs (24.4% of the sites) and cefuroxime for humans (12.8% of the sites).

### Important Antibiotics

Regarding this category, the offer is very low. As can be seen in Tables 3–5, Supplementary Tables S1–S3, only two antibiotics were available for purchase online: fusidic acid (4.7% of the sites in English and 2.6% of the sites in Spanish) that was offered for topical (4.7% of the sites in English and 1.3% of the sites in Spanish) and oral administration (0.8% of the sites in English and 1.3% of the sites in Spanish), and all formulations were intended for humans; thiostrepton (1.6% of the sites in English and 2.6% of the sites in Spanish) was sold only for the topical route and for cats and dogs.

## Discussion

In this study, we have carried out an observation at a point in time of the online availability of the families and each individual antibiotic included in the OIE list of antimicrobial agents of veterinary importance (22), reviewing the offers according to the administration route and the species for which the antibiotics were intended. We found that the most frequently sold families were penicillins and tetracyclines and the individual antibiotics amoxicillin and doxycycline. The formulations available were most frequently for oral or parenteral administration and intended for humans and for cats and dogs in English and for cats and dogs and for bovine in Spanish.

The most frequently offered families in the websites analyzed in English and in Spanish belonged to the category considered as “veterinary critically important” by the OIE (22). This is in line with antibiotic sale trends as reported by different governmental organizations. In 2019, in the UK, the most commonly sold classes of antibiotics were tetracyclines (32% of total sales) and penicillins (28% of total sales), although fluoroquinolones represented a low percentage (0.4%) (29). The same trend was observed in that year in food-producing animals in the USA, with tetracyclines and penicillins accounting for 67 and 12% of domestic sales, respectively, and with fluoroquinolones under 1% (30). As for the UE, although data are corrected by population (mg/PCU), tetracyclines and penicillins also accounted for 59.5% of sales in food-producing animals in 2018 (30.7 and 28.8%, respectively). Penicillins are also the most commonly sold pharmacological group in companion animals in the EU (30), and beta-lactams were the most commonly sold drugs for dogs and cats in the UK (76% of total sales) (29).

Regarding individual antibiotics, the high supply of certain specific compounds in the websites should also be highlighted. Amoxicillin and doxycycline were the most commonly offered compounds, both in English and in Spanish, and belong to the critically important category. In the case of amoxicillin, it is one of the most popular penicillins due to its broad spectrum of activity, and consequently, it is commonly used in the treatment of a variety of infectious diseases in domestic animals. In the tenth ESVAC report (31), it is stated that broad-spectrum penicillins (mainly represented by amoxicillin) accounted for the major proportion of penicillin sale for countries other than the Nordics and Switzerland. Doxycycline is the most frequently found tetracycline, probably related to its more favorable pharmacokinetic properties and, consequently, higher antimicrobial activity than the other tetracyclines. In a study carried out in Germany, it was observed that doxycycline was onwards the antibiotic that showed the largest sale volumes in the family of tetracyclines (32).

For some of these compounds, high levels of resistance in 2019 have been reported in *Escherichia coli* isolates from animal origin, such as neomycin (17.4%), amoxicillin/clavulanate (20.5%), trimethoprim/sulphonamide (38.2%), doxycycline (44.4%), and tetracycline (58.8%) in the UK (33). High-level resistance to ciprofloxacin was also exhibited by *Salmonella* Kentucky or *Campylobacter coli* isolates from poultry in 2018 (34). One of the biggest threats to global health today is antibiotic resistance, leading to higher treatment costs, longer hospital stay, and increased mortality. Antibiotic resistance is increasing to riskily high levels in all parts of the world. New resistance mechanisms are developing and spreading all around the world, threatening our possibilities to treat common infectious diseases (33). The use of these compounds in animals can contribute to the appearance of resistant bacteria that can be transferred to humans through the food chain or direct contact. This can diminish the effectiveness of antibiotics for treating human diseases (35). Pets are also involved in the generation of resistance to antimicrobials. Several studies have shown that companion animals are latent sources of spread of antimicrobial resistance due to the wide use of these drugs in these animals and their close contact with humans. Increasing trends of resistance to β-lactams and fluoroquinolones have been observed in most bacterial species of veterinary importance with zoonotic potential (36, 37). The control of antibiotic resistance is a high priority for WHO as well as for the European Medicines Agency (20, 38). The promotion of the appropriate use of antimicrobials in animals and the identification of the risk factors that may lead to the development and spread of antimicrobial resistance are essential in developing and monitoring policies on responsible use of these compounds.

Regarding the route of administration, we found that the most frequent formulations available were for oral or parenteral administration. The report UK-VARSS 2019 (29) showed that, in the UK, the most commonly sold antibiotics, taking into account the route of administration, were for oral and injectable administration. In the same way, the tenth ESVAC report (31) stated that the most commonly sold pharmaceutical forms for food-producing animals were for oral administration.

In this study, excluding humans, we found that the antibiotic offered was mainly for cats and dogs and for bovine. In the above-mentioned reports (29, 31), the data showed that the highest quantities of antibiotics sold were for pigs and for cattle. Companion animals were in the third place in the UK report (29), and the ESVAC report (31) did not include them. In this study, we could see that there are no important changes in the offer of antibiotics when comparing with a previous study (2). Around 80% of the web pages in English and in Spanish continued to sell antibiotics, and there were few changes in the requirement of prescription or the countries they sold from or to.

For this research, we created and used a web harvester. Our crawler is focused (it does not just exhaustively follow links, but it also searches for specific information, topics, and domains) and works in parallel (can operate on clusters of computers and/or multi-processor computers for maximum performance), allowing for fast online syntactic analysis of websites. Through a search engine (Google or Bing), a web search for specific terms was it performed, the URL found was extracted, and a first basic validation of them (removing ads, duplicated sites, uninteresting domains … and confirming the sites that in fact contain the terms we were looking for) was made, using the improved technology we developed in (24). Then, using an improved functionality that we developed for our current study, it further studied each validated website one by one, looking for specific words in each of them, whether we had explicitly searched for them (e.g., “buy antibiotics”) or not (e.g., antibiotic names and families).

After all the improvements of our core technology (24), we neither have to download the websites to study nor rely on third-party software (Heritrix) since we can now access and study each site “on the fly” as the spider visits them (online, without downloading them), which is faster, more reliable, and gets us fresh and updated information. Once the crawler was developed, tested, and ready to use, we ran it following the aforementioned steps: we made it look for, mark, and validate websites matching the search “buy antibiotics online.” Then, the crawler visited all the gathered sites, looking for the specific antibiotic names and categories that we previously give in detail in Table 1. For each crawled domain, the software creates a new entry in a local database (in the current version, it adds a new line to an Excel sheet, although we have also added some basic SQL support), detailing the site domain, the presence of the words it looked for through the search engine, and whether it found each antibiotic name and category in that domain or not. If a specific antibiotic/family is found, the domain-internal link to the page (the URL) is also recorded; this serves two purposes: it keeps track of all the information and knowledge extracted, and it allows for human validation since we, the researchers, can check if the term was indeed present, and if the context was suitable for our study—i.e., if the site was really selling that type of antibiotic and not just mentioning it. Some disadvantages are present: when using online analysis of the websites instead of downloading them, if the website is no longer accessible (because it is removed or because it is down), it is much harder (even impossible) to later check it manually; human validation is necessary, and sometimes there are problems in analyzing large websites.

The risk involved in the sale of antibiotics by illicit online pharmacies or digital platforms has been recognized (39). In this sense, several studies have suggested the need for increased health communication, promotion, and education initiatives to better inform potential buyers about the dangers of acquiring antibiotics online as well as the need to establish sale control procedures (40, 41). This study can contribute to the knowledge of the online offer of antibiotics; however, it has some limitations. We have evaluated the online sale offer for each individual antibiotic and the families included in the OIE list of antimicrobial agents of veterinary importance (22); however, we cannot determine the quantities of these compounds that are purchased (with or without prescription). On the other hand, the websites were located at a point in time so as to control antibiotic sales online, such that these data should be periodically actualized due to online offers that change continually: some sites can disappear or new ones can be created. In addition, local pages selling in other languages may exist and have not been included in our search.

We can conclude that the online availability of veterinary antibiotics was very high. After the search was carried out, we could see that there is a higher offer of websites selling antibiotics in English than in Spanish. Taking into account the route of administration, the offer was similar in English and in Spanish, and considering the species they are intended for, the offer was greater in Spanish. The offer of families and individual antibiotics was similar in English and in Spanish. It was especially troubling that the web pages reviewed sold mainly critically important antibiotics, with a greater offer than that of highly important antibiotics and with a very low offer for those considered important. The most offered families were penicillins, tetracyclines, fluoroquinolones, and aminoglycosides for oral and parenteral administration. The species for which the highest offer was found were cats and dogs as well as bovine.

## Data Availability Statement

The original contributions presented in the study are included in the article/Supplementary material, further inquiries can be directed to the corresponding author.

## Author Contributions

JFG programmed and carried out the search of the web pages. MS, JJG, MD, AS, RD, CL, and MF reviewed and evaluated the web pages selected to collect the information. JFG, MS, and MF analyzed the results obtained, drafted the manuscript, and coordinated the research. JJG carried out the statistical analysis and revised the manuscript. All authors read and approved the final manuscript.

## Conflict of Interest

The authors declare that the research was conducted in the absence of any commercial or financial relationships that could be construed as a potential conflict of interest.

## Publisher's Note

All claims expressed in this article are solely those of the authors and do not necessarily represent those of their affiliated organizations, or those of the publisher, the editors and the reviewers. Any product that may be evaluated in this article, or claim that may be made by its manufacturer, is not guaranteed or endorsed by the publisher.
