# Supplementary material for: Availability of Antibiotics for Veterinary Use on the Internet: A Cross-Sectional Study
Source: Front Vet Sci. 2022 Feb 11;8:798850. doi: 10.3389/fvets.2021.798850 (PMC8873107; doi:10.3389/fvets.2021.798850)
Supplement: Supplementary file 1 [file Data_Sheet_1.docx]

Supplementary Material

# Supplementary Data

**The Mirkwood crawler**

Mirkwood is composed of six modules: Seed Extraction, Environment, Forest, Spider, Spider Nest, and Spider Leg. Seed Extraction module is self-contained (it is also an autonomous piece of software, that is, it is able to work independently from the whole crawler). Figure 1 shows the architecture of the whole system. This image requires zoom in for its text to be properly visible, so it is only intended for the online version of this manuscript. A full resolution copy of this image is also available at (23). In the following, we describe all crawler modules in detail.

***1. Seed Extraction module***

This module feeds the Environment module with the URL of the hosts to crawl (seeds). It is self-contained (autonomous), so it can also be run as a standalone application or as a complement to any other crawler.

Seeds are obtained in three consecutive steps: Access to the search engine, search, and URL extraction. These steps can be performed either manually or automatically; to achieve automation, the extraction module uses the third party software tool Selenium WebDriver (24).

**Access to the Search Engine.-** We use Google or Bing, accessed via the browser Firefox or Google Chrome, respectively. The reason for using one or the other has been to take advantage of other existing software tools which are engine and browser-specific, as well as getting more variety for the seeds gathered.

**Search.-** Our software can collect thousands of seeds from a single search query. Since the seed extraction module relies on the aforementioned search engines, it can perform both simple and complex searches (based on patterns and operators).

**URL extraction.-** To comply with terms of service, we collect URL directly from the engine interface in the browser (which is known as SERP, Search Engine Results Page) using Grease monkey and an Internet Marketing Ninjas plug-in and an ad-hoc JavaScript plug-in (25). The quality of seeds obtained is improved by reducing both human fatigue and propensity for human error, and by automatically discarding ads and fraudulent sites

This module was the technological base for our study on online sale of antibiotics for veterinary use (18) and is extensively detailed in both (20) and (18). Figure 2 depicts both Seed Extraction and Environment modules.

***2. Environment module***

Environment module runs in the main machine, reading configuration files and the list of initial hosts to crawl, which gets from Seed extraction module output. This module can also get the URL list from an external text file provided by a user. We relied on the latter functionality to inject the same domains we initially extracted and checked in (18). Some of the most relevant parameters set up by this module are the analysis terms (terms to look for at the different domains, in this case, the whole list of antibiotics which is presented at section 4.2.5.), the search patterns to perform the searches, and the output directory where the crawler will store its results. All this information is sent to the Forest module.

***3. Forest module***

Forest is the main module of Mirkwood. It sets up and starts the crawling process according to the information received from the Environment module. The Forest module creates a set of Spider Nests to which it distributes the target seeds. All communication between the Forest module and each Spider Nest is done via MPI (22). Spider Nests can not communicate with each other directly. A Spider Nest is created for each machine available in the computer system which is running the crawler, so we can have any amount of nests from 1 to N, N being the amount of available machines.

Once the Spider Nests have been created, the Forest module distributes the websites to crawl among them. A lightweight analysis of machine specifications and websites complexity is performed to try to optimize load balance among them. In Figure 1 and Figure 3, these sets of pages sent to a specific machine are labelled as “Website set n” and shortened as “WSn”. The Forest module periodically receives analysis results from its Nests every time a Spider finishes crawling a full domain. Upon receiving a report, the Forest module updates both the crawling results file and the site map of the domains that are being analysed.

***4. Spider Nest module***

Spider Nest functionality encompasses Spiders creation and synchronization, and report–forwarding from Spiders to the Forest: every time a report message is received from a Spider, it is immediately sent to the Forest module. There is one (and just one) Spider Nest in every machine (physical or virtual). The set of pages to crawl is received from the Forest. This set (labelled as “Website set n” and shortened as “WSn” in Figure 1 and Figure 4) is split into subsets which are distributed between Spiders, one subset for each Spider (these subsets are labelled as “WSn−subset m”– WSn identifies the package split in Forest and received at the Nest– and are abbreviated as “WSn−Sm” in Figures 1 and 4). As previously noted, Spider Nest communication with the Forest is achieved via MPI. Information flow between each Nest to its Spiders is enabled through Threading. This is the natural implementation of the communication system considering all Spiders “living” in a specific Nest are running in the same machine of that Nest (remember, we have one Nest for each machine), while each Nest is located in a separate machine: On the one hand, communication is guaranteed among threads of the same process (by thread definition and implementation); on the other hand, MPI technology was designed specifically to communicate processes in different machines.

***5. Spider and Spider Legs modules***

Spider module gets the list of domains (websites) to crawl from its Nest. Each Spider goes over this list of hosts, crawling them one by one. Unless configured by the user, Spiders visit only links internal to these domains (Spiders do not “jump” outside the domains). The crawling process itself is performed by Spider Legs. Spiders have no legs when they are created. For every URL (starting at the domains’ home page), the Spider spawns a Spider Leg, which analyses its content and extracts all links it contains; every time a given URL is fully analysed, the leg which took care of it is de–spawned. The crawling process for each Spider continues until it has no legs left, which means the domain has been fully crawled. As soon as a site is fully crawled, the Spider sends a site final report to its parent Nest and goes on with the next domain (if any).

#
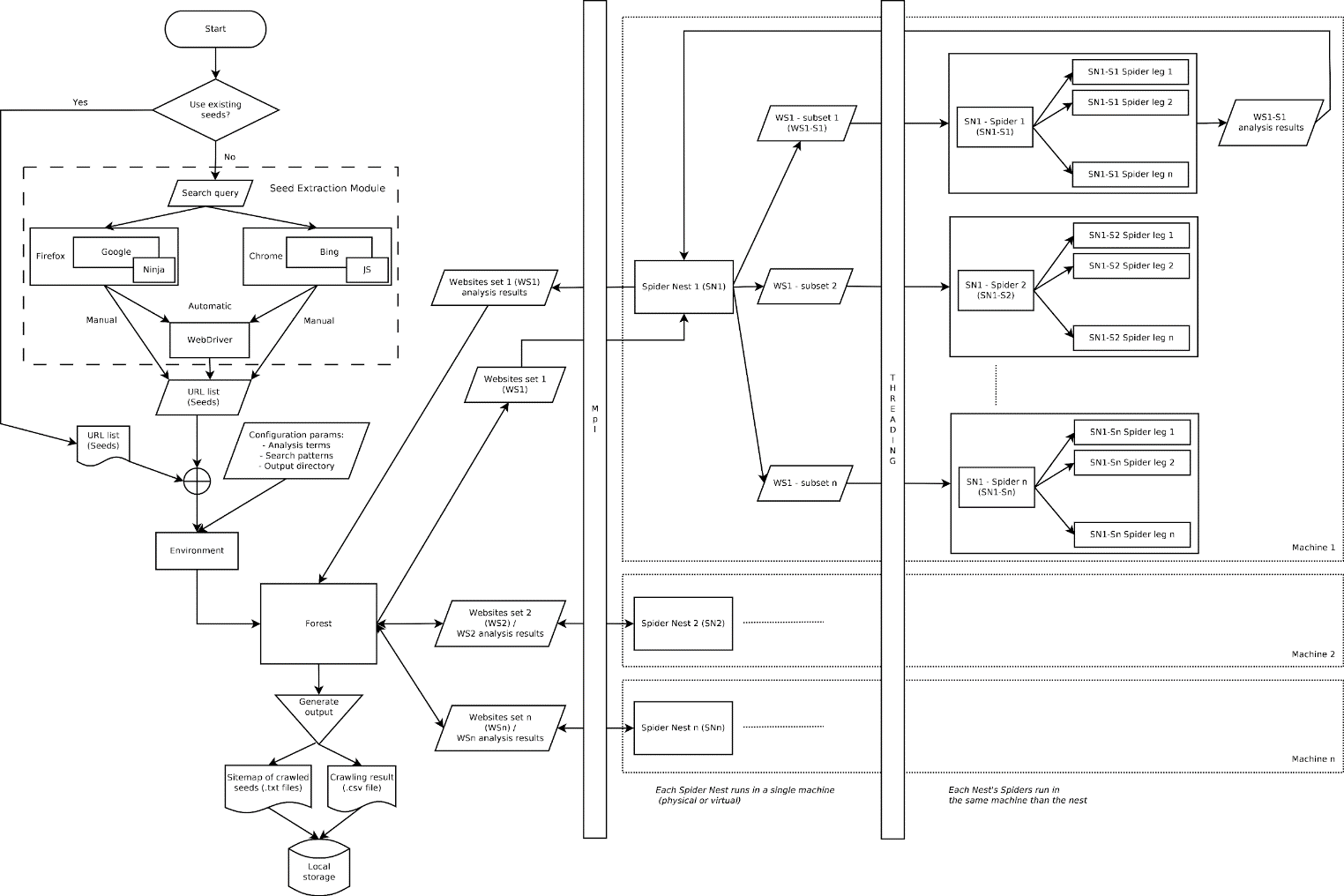


**Figure 1.** Mirkwood architecture.


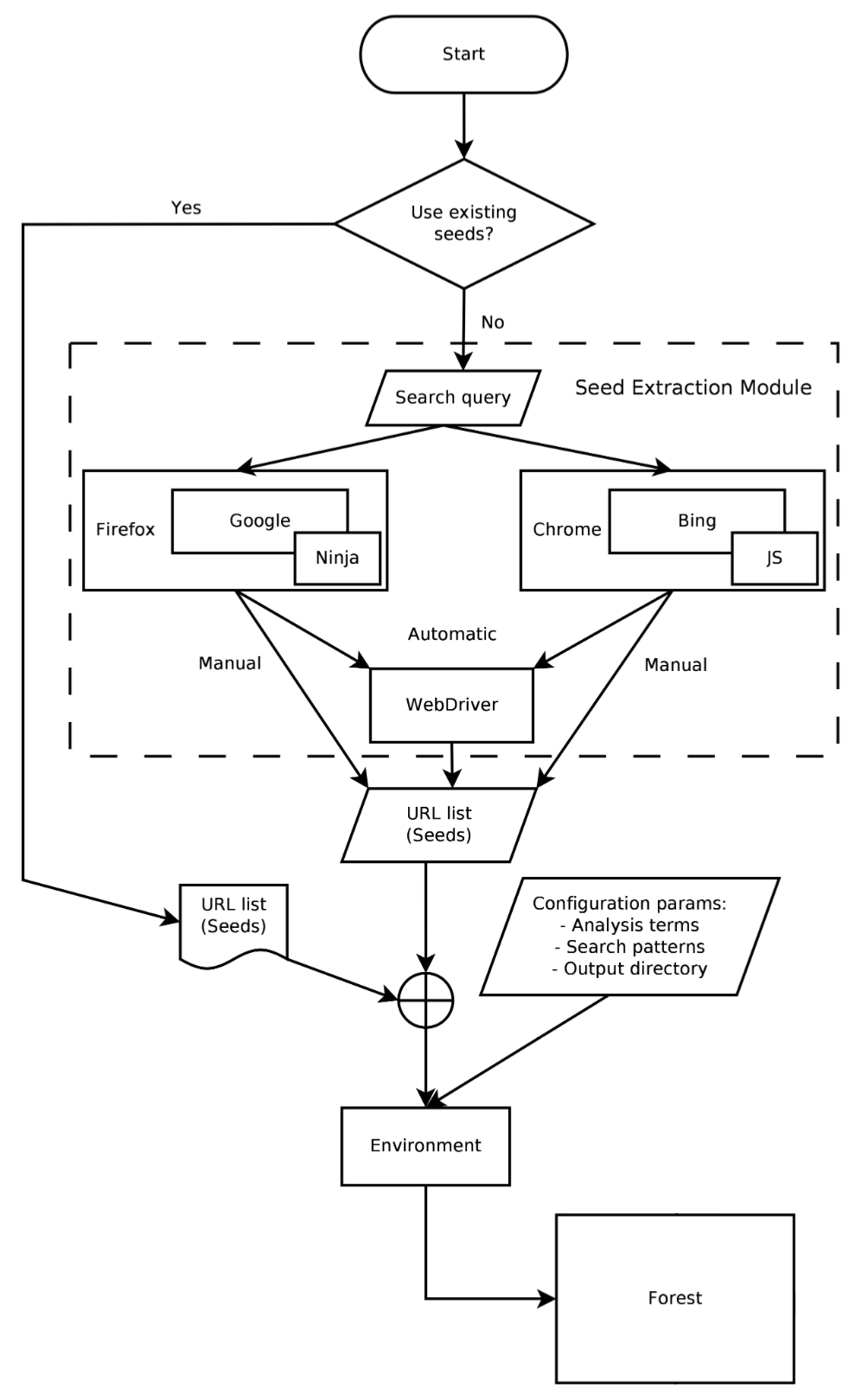


**Figure 2.** Seed Extraction and Environment modules [detail].


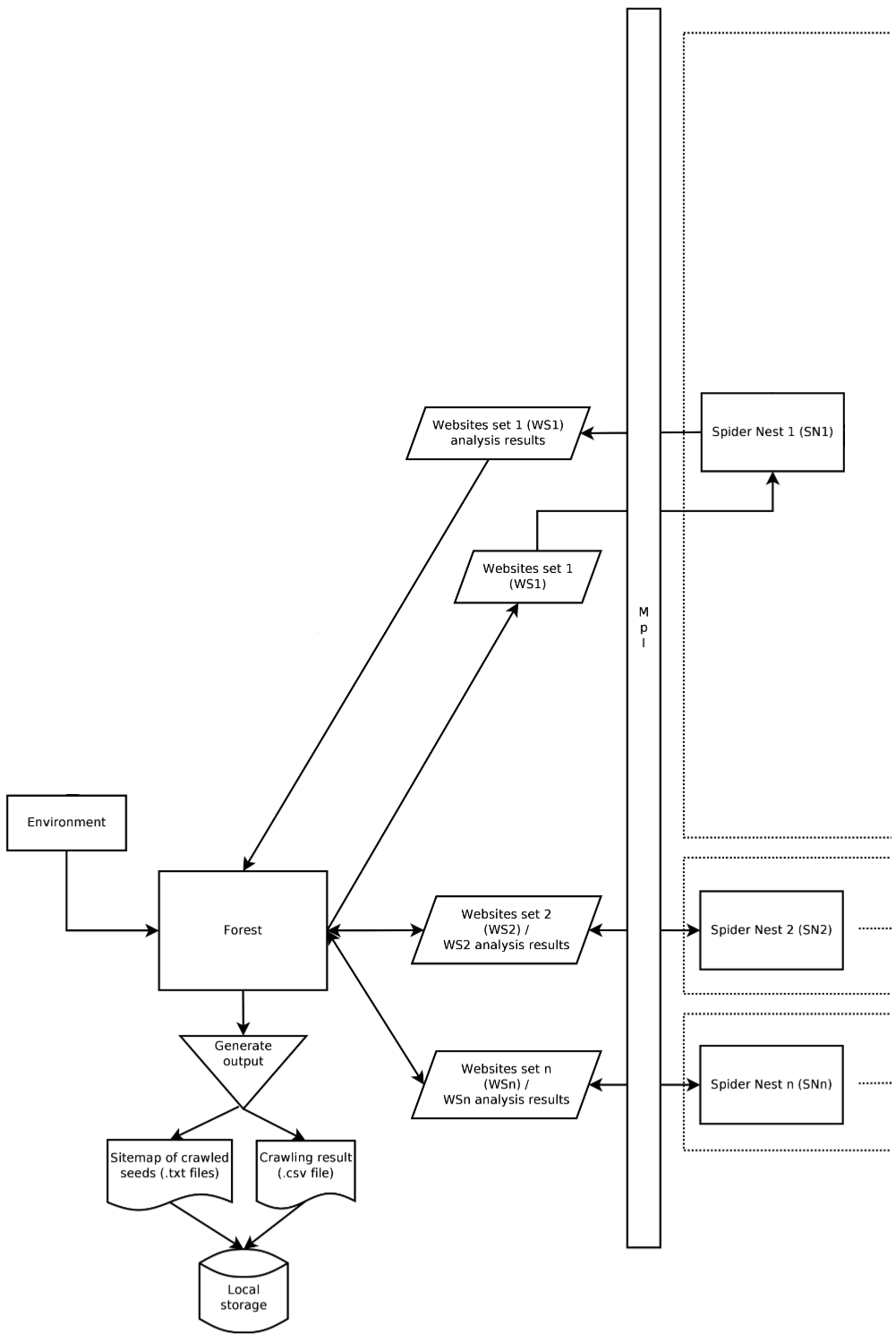


**Figure 3.** Forest module [detail]


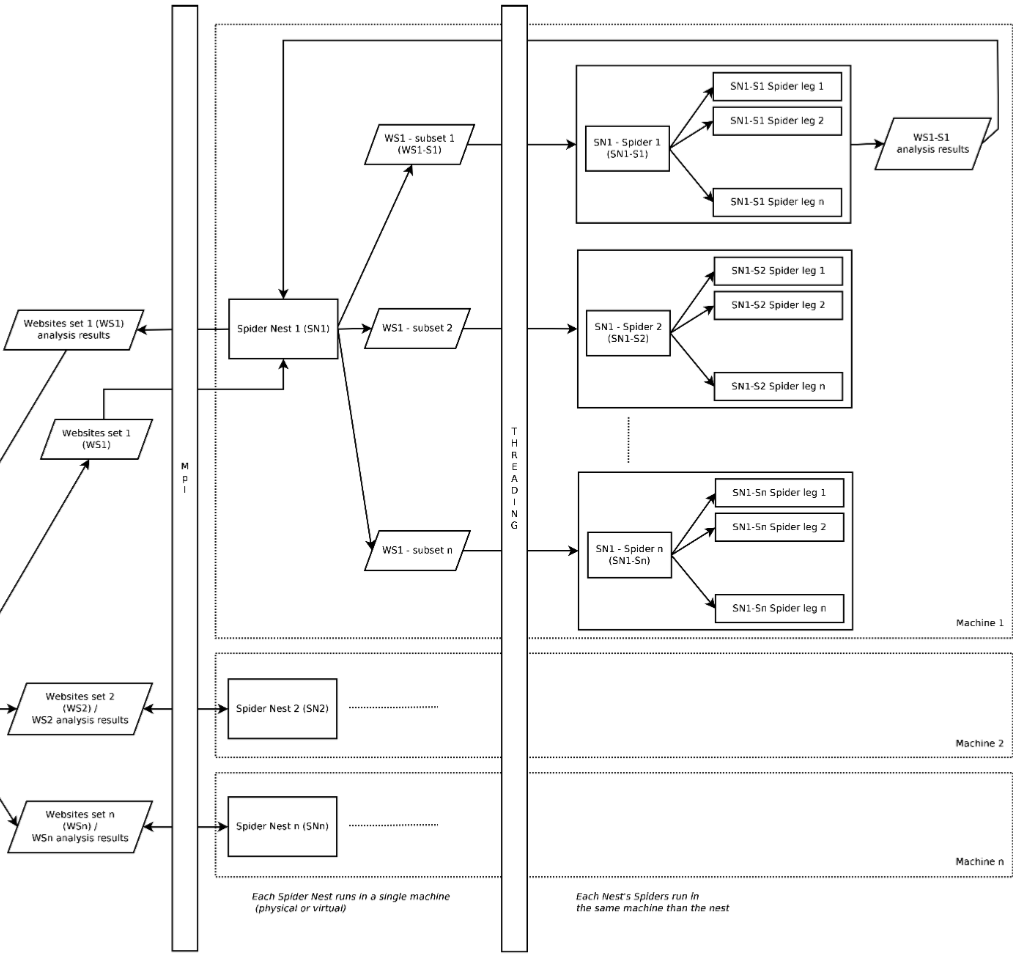


**Figure 4.** Spider Nest, Spider, and Spider Legs modules [detail].

**STROBE CHECKLIST**

STROBE Statement—Checklist of items that should be included in reports of ***cross-sectional studies***

|  | Item No | Recommendation | Page No |
| --- | --- | --- | --- |
| **Title and abstract** | 1 | (*a*) Indicate the study’s design with a commonly used term in the title or the abstract | 1 |
|  |  | (*b*) Provide in the abstract an informative and balanced summary of what was done and what was found | 1 lines 13-26 |
| Introduction | | | |
| Background/rationale | 2 | Explain the scientific background and rationale for the investigation being reported | 1-2 lines 29-57 |
| Objectives | 3 | State specific objectives, including any prespecified hypotheses | 2 lines 58-61 |
| Methods | | | |
| Study design | 4 | Present key elements of study design early in the paper | 2 lines 72-75 |
| Setting | 5 | Describe the setting, locations, and relevant dates, including periods of recruitment, exposure, follow-up, and data collection | 2 lines 64-70 |
| Participants | 6 | (*a*) Give the eligibility criteria, and the sources and methods of selection of participants | N/A |
| Variables | 7 | Clearly define all outcomes, exposures, predictors, potential confounders, and effect modifiers. Give diagnostic criteria, if applicable | N/A |
| Data sources/ measurement | 8* | For each variable of interest, give sources of data and details of methods of assessment (measurement). Describe comparability of assessment methods if there is more than one group | 3 lines 78-93 |
| Bias | 9 | Describe any efforts to address potential sources of bias | N/A |
| Study size | 10 | Explain how the study size was arrived at | N/A |
| Quantitative variables | 11 | Explain how quantitative variables were handled in the analyses. If applicable, describe which groupings were chosen and why | N/A |
| Statistical methods | 12 | (*a*) Describe all statistical methods, including those used to control for confounding | N/A |
|  |  | (*b*) Describe any methods used to examine subgroups and interactions | 3-4 lines 95-130 |
|  |  | (*c*) Explain how missing data were addressed | N/A |
|  |  | (*d*) If applicable, describe analytical methods taking account of sampling strategy | N/A |
|  |  | (*e*) Describe any sensitivity analyses | N/A |
| Results | | | |
| Participants | 13* | (a) Report numbers of individuals at each stage of study—eg numbers potentially eligible, examined for eligibility, confirmed eligible, included in the study, completing follow-up, and analysed | 4 lines 132-133 |
|  |  | (b) Give reasons for non-participation at each stage | 4 lines 135-138 |
|  |  | (c) Consider use of a flow diagram |  |
| Descriptive data | 14* | (a) Give characteristics of study participants (eg demographic, clinical, social) and information on exposures and potential confounders | 4-5 lines 153-165 |
|  |  | (b) Indicate number of participants with missing data for each variable of interest | N/A |
| Outcome data | 15* | Report numbers of outcome events or summary measures | 5-6 lines 169-229 |
| Main results | 16 | (*a*) Give unadjusted estimates and, if applicable, confounder-adjusted estimates and their precision (eg, 95% confidence interval). Make clear which confounders were adjusted for and why they were included | N/A |
|  |  | (*b*) Report category boundaries when continuous variables were categorized | N/A |
|  |  | (*c*) If relevant, consider translating estimates of relative risk into absolute risk for a meaningful time period | N/A |
| Other analyses | 17 | Report other analyses done—eg analyses of subgroups and interactions, and sensitivity analyses | N/A |
| Discussion | | | |
| Key results | 18 | Summarise key results with reference to study objectives | 6 lines 231-237 |
| Limitations | 19 | Discuss limitations of the study, taking into account sources of potential bias or imprecision. Discuss both direction and magnitude of any potential bias | 8 lines 324-331 |
| Interpretation | 20 | Give a cautious overall interpretation of results considering objectives, limitations, multiplicity of analyses, results from similar studies, and other relevant evidence | 6-8 lines 238-323 |
| Generalisability | 21 | Discuss the generalisability (external validity) of the study results | N/A |
| Other information | | | |
| Funding | 22 | Give the source of funding and the role of the funders for the present study and, if applicable, for the original study on which the present article is based | The study was not funded |

# Supplementary Figures and Tables

**Table S1**: Number of websites where the individual antibiotics can be purchased, classified following the OIE list of antimicrobial agents of veterinary importance (22), in the searches in English and in Spanish

|  | **English (n = 129)** | **Spanish (n = 78)** | **Total** |
| --- | --- | --- | --- |
| **CRITICALLY IMPORTANT** | | | |
| **AMINOGLYCOSIDES** | **37** | **45** | **82** |
| Aminocyclitol |  |  |  |
| Amikacin | 7 | 4 | 11 |
| Apramycin |  |  |  |
| Dihydrostreptomycin | 2 | 23 | 25 |
| Fortimycin |  |  |  |
| Framycetin | 1 | 3 | 4 |
| Gentamicin | 22 | 36 | 58 |
| Kanamycin | 2 | 5 | 7 |
| Neomycin | 29 | 30 | 59 |
| Paromomycin |  |  |  |
| Spectinomycin | 8 | 7 | 15 |
| Streptomycin | 2 | 15 | 17 |
| Tobramycin | 12 | 13 | 25 |
| **AMPHENICOLS** | **5** | **16** | **21** |
| Florphenicol | 5 | 16 | 21 |
| Thiamphenicol |  | 1 | 1 |
| **CEPHALOSPORINS** | **11** | **20** | **31** |
| Cefoperazone | 1 |  | 1 |
| Ceftiofur | 6 | 16 | 22 |
| Ceftriaxone | 5 | 3 | 8 |
| Cefquinoma |  | 4 | 4 |
| **MACROLIDES** | **57** | **40** | **97** |
| Carbomycin |  |  |  |
| Erythromycin | 46 | 18 | 64 |
| Gamithromycin | 1 | 1 | 2 |
| Josamycin |  |  |  |
| Kitasamycin |  | 1 | 1 |
| Mirosamycin |  |  |  |
| Oleandomycin | 3 |  | 3 |
| Sedecamycin |  |  |  |
| Spiramycin | 2 | 25 | 27 |
| Terdecamycin |  |  |  |
| Tildipirosin | 3 |  | 3 |
| Tilmicosin | 2 | 8 | 10 |
| Tulathromycin | 4 | 4 | 8 |
| Tylosin | 15 | 13 | 28 |
| Tylvalosin | 2 |  | 2 |
| **PENICILLINS** | **72** | **51** | **123** |
| **Natural Penicillins** | 21 | 30 | 51 |
| Benethamine penicillin | 1 | 1 | 2 |
| Benzylpenicillin | 7 | 16 | 23 |
| Penethamate |  | 3 | 3 |
| Bencilpenicillin procaine | 13 | 19 | 32 |
| Benzatine penicillin | 7 | 20 | 27 |
| **Amdinopenicillins** |  |  |  |
| Mecillinam |  |  |  |
| **Aminopenicillins** | 66 | 45 | 111 |
| Amoxicillin | 64 | 45 | 109 |
| Amoxicillin/Clavulanic Acid | 44 | 24 | 68 |
| Ampicillin | 41 | 28 | 69 |
| Ampicillin/Sulbactam | 4 | 1 | 5 |
| Hetacillin | 1 | 0 | 1 |
| **Carboxypenicillins** |  |  |  |
| Ticarcillin |  |  |  |
| Tobicillin |  |  |  |
| **Ureidopenicillin** |  |  |  |
| Aspoxicillin |  |  |  |

**Table S1 (continuation)**: Number of websites where the individual antibiotics can be purchased, classified following the OIE list of antimicrobial agents of veterinary importance (22), in the searches in English and in Spanish

|  | **English (n = 129)** | **Spanish (n = 78)** | **Total** |
| --- | --- | --- | --- |
| **CRITICALLY IMPORTANT** | | | |
| **Phenoxypenicillins** | 6 | 2 | 8 |
| Phenoxymethylpenicillin | 6 | 2 | 8 |
| Phenethicillin |  |  |  |
| **Antistaphylococcal Penicillins** | 10 | 15 | 25 |
| Cloxacillin | 8 | 14 | 22 |
| Dicloxacillin | 3 | 1 | 4 |
| Nafcillin | 3 | 1 | 4 |
| Oxacillin | 2 |  | 2 |
| **FLUOROQUINOLONES** | **68** | **53** | **121** |
| Ciprofloxacin | 55 | 24 | 79 |
| Danofloxacin | 2 | 3 | 5 |
| Difloxacin | 1 | 1 | 2 |
| Enrofloxacin | 20 | 42 | 62 |
| Marbofloxacin | 10 | 7 | 17 |
| Norfloxacin | 22 | 24 | 46 |
| Ofloxacin | 30 | 9 | 39 |
| Orbifloxacin | 6 | 1 | 7 |
| Sarafloxacin |  |  |  |
| **SULFONAMIDES** | **35** | **41** | **76** |
| Phthalylsulfathiazole |  | 6 | 6 |
| Sulfachlorpyridazine | 1 | 1 | 2 |
| Sulfadiazine | 14 | 22 | 36 |
| Sulfadimerazin | 1 |  | 1 |
| Sulfadimethoxazole | 3 | 10 | 13 |
| Sulfadimethoxine | 9 | 10 | 19 |
| Sulfadimidine | 5 |  | 5 |
| Sulfadoxine | 3 | 12 | 15 |
| Sulfafurazole |  |  |  |
| Sulfaguanidine |  | 7 | 7 |
| Sulfamerazine |  | 1 | 1 |
| Sulfamethazine | 5 | 9 | 14 |
| Sulfamethoxine |  | 2 | 2 |
| Sulfamethoxypyridazine | 1 | 3 | 4 |
| Sulfamonomethoxine |  | 1 | 1 |
| Sulfanilamide | 1 | 6 | 7 |
| Sulfapyridine |  |  |  |
| Sulfaquinoxaline | 1 | 7 | 8 |
| **DIAMINOPYRIMIDINES** | **9** | **6** | **15** |
| Baquiloprim |  |  |  |
| Trimethoprim | 9 | 5 | 14 |
| Ormetoprim |  | 1 | 1 |
| **SULFONAMIDES + DIAMINOPYRIMIDINES** | **50** | **26** | **76** |
| Sulfadimethoxine/Ormetoprim | 49 | 23 | 72 |
| Sulfonamide/Trimethoprim | 1 | 3 | 4 |
| **TETRACYCLINES** | **71** | **53** | **124** |
| Chlortetracycline | 7 | 11 | 18 |
| Doxycycline | 60 | 42 | 102 |
| Oxytetracycline | 31 | 23 | 54 |
| Tetracycline | 36 | 17 | 53 |

**Table S1 (continuation)**: Number of websites where the individual antibiotics can be purchased, classified following the OIE list of antimicrobial agents of veterinary importance (22), in the searches in English and in Spanish

|  | **English (n = 129)** | **Spanish (n = 78)** | **Total** |
| --- | --- | --- | --- |
| **HIGHLY IMPORTANT** | | | |
| **ANSAMYCIN – RIFAMYCINS** | **14** | **7** | **21** |
| Ansamycin |  |  |  |
| Rifampicin | 7 |  | 7 |
| Rifaximin | 11 | 7 | 18 |
| **CEPHALOSPORINS** | **56** | **36** | **92** |
| Cefacetrile |  | 2 | 2 |
| Cefalexin | 51 | 32 | 83 |
| Cefalotin |  | 1 | 1 |
| Cefapyrin | 1 | 1 | 2 |
| Cefazolin | 4 | 1 | 5 |
| Cefalonium | 1 | 1 | 2 |
| Cefuroxime | 24 | 10 | 34 |
| **IONOPHORES** |  | **1** | **1** |
| Lasalocid |  |  |  |
| Maduramycin |  |  |  |
| Monensin |  |  |  |
| Narasin |  |  |  |
| Salinomycin |  | 1 | 1 |
| Semduramicin |  |  |  |
| **LINCOSAMIDES** | **29** | **19** | **48** |
| Pirlimycin | 1 |  | 1 |
| Lincomycin | 28 | 19 | 47 |
| **PHOSPHONIC ACID** | **1** | **3** | **4** |
| Phosphonic Acid |  |  |  |
| Fosfomycin | 1 | 3 | 4 |
| **PLEUROMUTILINS** | **3** | **4** | **7** |
| Tiamulin | 3 | 4 | 7 |
| Valnemulin |  |  |  |
| **POLYPEPTIDES** | **23** | **19** | **42** |
| Enramycin |  |  |  |
| Gramicidin | 1 | 3 | 4 |
| Bacitracin | 17 | 14 | 31 |
| Colistin | 2 | 5 | 7 |
| Polymixin | 22 | 12 | 34 |
| **QUINOLONES** | **6** | **3** | **9** |
| Flumequin | 1 |  | 1 |
| Miloxacin |  |  |  |
| Nalidixic acid | 6 | 3 | 9 |
| Oxolinic acid |  |  |  |

**Table S1 (continuation)**: Number of websites where the individual antibiotics can be purchased, classified following the OIE list of antimicrobial agents of veterinary importance (22), in the searches in English and in Spanish

|  | **English (n = 129)** | **Spanish (n = 78)** | **Total** |
| --- | --- | --- | --- |
| **IMPORTANT** | | | |
| **AMINOCOUMARIN** |  |  |  |
| Aminocoumarin |  |  |  |
| Novobiocin |  |  |  |
| **ARSENICAL** |  |  |  |
| Roxarsone |  |  |  |
| Nitarsone |  |  |  |
| **BICYCLOMYCIN** |  |  |  |
| Bicyclomycin |  |  |  |
| Bicozamycin |  |  |  |
| **FUSIDIC ACID** | **6** | **2** | **8** |
| Fusidic acid | 6 | 2 | 8 |
| **ORTHOSOMYCINS** |  |  |  |
| Avilamycin |  |  |  |
| **QUINOXALINES** |  |  |  |
| Carbadox |  |  |  |
| Olaquindox |  |  |  |
| **STREPTOGRAMINS** |  |  |  |
| Virginiamycin |  |  |  |
| **THIOSTREPTON** | **2** | **2** | **4** |
| Thiostrepton | 2 | 2 | 4 |
| Nosiheptide |  |  |  |

**Table S2**: Number of websites where the individual antibiotics can be purchased according to the administration route, classified following the OIE list of antimicrobial agents of veterinary importance (22), in the searches in English and in Spanish

|  | **Oral** | | | **Parenteral** | | | **Topic** | | | **Intramammary** | | | **Intrauterin** | | | **Unknown** | | |
| --- | --- | --- | --- | --- | --- | --- | --- | --- | --- | --- | --- | --- | --- | --- | --- | --- | --- | --- |
|  | **E** | **S** | **T** | **E** | **S** | **T** | **E** | **S** | **T** | **E** | **S** | **T** | **E** | **S** | **T** | **E** | **S** | **T** |
| **CRITICALLY IMPORTANT** | | | | | | | | | | | | | | | | | | |
| **AMINOGLYCOSIDES** | **27** | **18** | **45** | **20** | **68** | **88** | **36** | **61** | **97** | **2** | **21** | **23** | **1** | **4** | **5** | **7** |  | **7** |
| Aminocyclitol |  |  |  |  |  |  |  |  |  |  |  |  |  |  |  |  |  |  |
| Amikacin | 1 | 1 | 2 | 5 | 3 | 8 |  | 1 | 1 |  |  |  |  |  |  | 1 |  | 1 |
| Apramycin |  |  |  |  |  |  |  |  |  |  |  |  |  |  |  |  |  |  |
| Dihydrostreptomycin |  |  |  | 1 | 20 | 21 |  | 3 | 3 | 1 | 2 | 3 |  |  |  |  |  |  |
| Fortimycin |  |  |  |  |  |  |  |  |  |  |  |  |  |  |  |  |  |  |
| Framycetin |  |  |  |  |  |  | 1 | 2 | 3 |  | 1 | 1 |  |  |  |  |  |  |
| Gentamicin | 4 | 4 | 8 | 8 | 21 | 29 | 12 | 21 | 33 |  | 5 | 5 | 1 | 3 | 4 | 2 |  | 2 |
| Kanamycin | 1 |  | 1 |  | 3 | 3 |  | 2 | 2 |  | 2 | 2 |  |  |  | 1 |  | 1 |
| Neomycin | 12 | 7 | 19 | 2 | 2 | 4 | 15 | 19 | 34 | 1 | 11 | 12 |  | 1 | 1 | 1 |  | 1 |
| Paromomycin |  |  |  |  |  |  |  |  |  |  |  |  |  |  |  |  |  |  |
| Spectinomycin | 7 | 4 | 11 | 1 | 5 | 6 |  |  |  |  |  |  |  |  |  |  |  |  |
| Streptomycin |  | 1 | 1 | 1 | 14 | 15 |  | 1 | 1 |  |  |  |  |  |  | 1 |  | 1 |
| Tobramycin | 2 | 1 | 3 | 2 |  | 2 | 8 | 12 | 20 |  |  |  |  |  |  | 1 |  | 1 |
| **AMPHENICOLS** |  | **9** | **9** | **4** | **11** | **15** |  | **1** | **1** |  |  |  |  |  |  | **1** |  | **1** |
| Florphenicol |  | 8 | 8 | 4 | 10 | 14 |  | 1 | 1 |  |  |  |  |  |  | 1 |  | 1 |
| Thiamphenicol |  | 1 | 1 |  | 1 | 1 |  | 0 | 0 |  |  |  |  |  |  |  |  |  |
| **CEPHALOSPORINS** | **3** |  | **3** | **9** | **23** | **32** |  | **1** | **1** | **2** | **2** | **4** |  |  |  |  |  |  |
| Cefoperazone | 1 |  | 1 | 1 |  | 1 |  |  |  |  |  |  |  |  |  |  |  |  |
| Ceftiofur |  |  |  | 6 | 16 | 22 |  |  |  | 1 | 1 | 2 |  |  |  |  |  |  |
| Ceftriaxone | 2 |  | 2 | 2 | 3 | 5 |  | 1 | 1 | 1 |  | 1 |  |  |  |  |  |  |
| Cefquinoma |  |  |  |  | 4 | 4 |  |  |  |  | 1 | 1 |  |  |  |  |  |  |
| **MACROLIDES** | **55** | **40** | **95** | **22** | **25** | **47** | **3** | **5** | **8** |  | **9** | **9** |  |  |  | **3** |  | **3** |
| Carbomycin |  |  |  |  |  |  |  |  |  |  |  |  |  |  |  |  |  |  |
| Erythromycin | 41 | 12 | 53 | 5 | 4 | 9 | 3 | 4 | 7 |  | 2 | 2 |  |  |  | 1 |  | 1 |
| Gamithromycin |  |  |  | 1 | 1 | 2 |  |  |  |  |  |  |  |  |  |  |  |  |
| Josamycin |  |  |  |  |  |  |  |  |  |  |  |  |  |  |  |  |  |  |
| Kitasamycin |  | 1 | 1 |  |  |  |  |  |  |  |  |  |  |  |  |  |  |  |
| Mirosamycin |  |  |  |  |  |  |  |  |  |  |  |  |  |  |  |  |  |  |
| Oleandomycin | 2 |  | 2 |  |  |  |  |  |  |  |  |  |  |  |  | 1 |  | 1 |
| Sedecamycin |  |  |  |  |  |  |  |  |  |  |  |  |  |  |  |  |  |  |
| Spiramycin | 2 | 17 | 19 |  | 1 | 1 |  |  |  |  | 7 | 7 |  |  |  |  |  |  |
| Terdecamycin |  |  |  |  |  |  |  |  |  |  |  |  |  |  |  |  |  |  |
| Tildipirosin |  |  |  | 3 |  | 3 |  |  |  |  |  |  |  |  |  |  |  |  |
| Tilmicosin |  | 2 | 2 | 2 | 7 | 9 |  |  |  |  |  |  |  |  |  |  |  |  |
| Tulathromycin |  |  |  | 4 | 4 | 8 |  |  |  |  |  |  |  |  |  |  |  |  |
| Tylosin | 8 | 8 | 16 | 7 | 8 | 15 |  | 1 | 1 |  |  |  |  |  |  | 1 |  | 1 |
| Tylvalosin | 2 |  | 2 |  |  |  |  |  |  |  |  |  |  |  |  |  |  |  |
| **PENICILLINS** | **165** | **83** | **248** | **36** | **85** | **121** | **1** | **6** | **7** | **5** | **25** | **30** |  | **2** | **2** | **2** |  | **2** |
| **Natural Penicillins** |  |  |  |  |  |  |  |  |  |  |  |  |  |  |  |  |  |  |
| Benethamine penicillin | 1 |  | 1 |  |  |  |  |  |  |  | 1 | 1 |  |  |  |  |  |  |
| Benzylpenicillin | 7 |  | 7 |  | 16 | 16 |  |  |  |  | 1 | 1 |  |  |  |  |  |  |
| Penethamate |  |  |  |  | 2 | 2 |  |  |  |  | 1 | 1 |  |  |  |  |  |  |
| Bencilpenicillin procaine |  |  |  | 13 | 18 | 31 |  | 3 | 3 | 1 |  | 1 |  | 1 | 1 |  |  |  |
| Benzatine penicillin |  |  |  | 7 | 20 | 27 |  | 2 | 2 |  | 1 | 1 |  |  |  |  |  |  |
| **Amdinopenicillins** |  |  |  |  |  |  |  |  |  |  |  |  |  |  |  |  |  |  |
| Mecillinam |  |  |  |  |  |  |  |  |  |  |  |  |  |  |  |  |  |  |
| **Aminopenicillins** |  |  |  |  |  |  |  |  |  |  |  |  |  |  |  |  |  |  |
| Amoxicillin | 62 | 39 | 101 | 3 | 11 | 14 | 1 |  | 1 |  | 5 | 5 |  |  |  | 1 |  | 1 |
| Amoxicillin/Clavulanic Acid | 44 | 24 | 68 |  | 1 | 1 |  |  |  |  | 2 | 2 |  |  |  |  |  |  |
| Ampicillin | 33 | 15 | 48 | 7 | 12 | 19 |  |  |  | 1 | 4 | 5 |  | 1 | 1 | 1 |  | 1 |
| Ampicillin/ Sulbactam |  |  |  | 4 | 1 | 5 |  |  |  |  |  |  |  |  |  |  |  |  |
| Hetacillin |  |  |  |  |  |  |  |  |  | 1 |  | 1 |  |  |  |  |  |  |
| **Carboxypenicillins** |  |  |  |  |  |  |  |  |  |  |  |  |  |  |  |  |  |  |
| Ticarcillin |  |  |  |  |  |  |  |  |  |  |  |  |  |  |  |  |  |  |
| Tobicillin |  |  |  |  |  |  |  |  |  |  |  |  |  |  |  |  |  |  |
| **Ureidopenicillin** |  |  |  |  |  |  |  |  |  |  |  |  |  |  |  |  |  |  |
| Aspoxicillin |  |  |  |  |  |  |  |  |  |  |  |  |  |  |  |  |  |  |

E.- English; S.- Spanish; T.- Total

**Table S2 (continuation)**: Number of websites where the individual antibiotics can be purchased according to the administration route, classified following the OIE list of antimicrobial agents of veterinary importance (22), in the searches in English and in Spanish

|  | **Oral** | | | **Parenteral** | | | **Topic** | | | **Intramammary** | | | **Intrauterin** | | | **Unknown** | | |
| --- | --- | --- | --- | --- | --- | --- | --- | --- | --- | --- | --- | --- | --- | --- | --- | --- | --- | --- |
|  | **E** | **S** | **T** | **E** | **S** | **T** | **E** | **S** | **T** | **E** | **S** | **T** | **E** | **S** | **T** | **E** | **S** | **T** |
| **Phenoxypenicillins** |  |  |  |  |  |  |  |  |  |  |  |  |  |  |  |  |  |  |
| Phenoxymethylpenicillin | 6 | 1 | 7 |  | 1 | 1 |  |  |  |  |  |  |  |  |  |  |  |  |
| Phenethicillin |  |  |  |  |  |  |  |  |  |  |  |  |  |  |  |  |  |  |
| **Antistaphylococcal Penicillins** |  |  |  |  |  |  |  |  |  |  |  |  |  |  |  |  |  |  |
| Cloxacillin | 6 | 3 | 9 |  | 3 | 3 |  | 1 | 1 | 2 | 9 | 11 |  |  |  |  |  |  |
| Dicloxacillin | 3 | 1 | 4 |  |  |  |  |  |  |  |  |  |  |  |  |  |  |  |
| Nafcillin | 2 |  | 2 | 1 |  | 1 |  |  |  |  | 1 | 1 |  |  |  |  |  |  |
| Oxacillin | 1 |  | 1 | 1 |  | 1 |  |  |  |  |  |  |  |  |  |  |  |  |
| **FLUOROQUINOLONES** | **128** | **80** | **208** | **13** | **23** | **36** | **24** | **31** | **55** |  | **1** | **1** |  |  |  | **7** |  | **7** |
| Ciprofloxacin | 51 | 15 | 66 | 2 |  | 2 | 9 | 11 | 20 |  | 1 | 1 |  |  |  | 2 |  | 2 |
| Danofloxacin |  |  |  | 2 | 3 | 5 |  |  |  |  |  |  |  |  |  |  |  |  |
| Difloxacin |  | 1 | 1 |  |  |  | 1 |  | 1 |  |  |  |  |  |  |  |  |  |
| Enrofloxacin | 14 | 34 | 48 | 7 | 17 | 24 | 4 | 12 | 16 |  |  |  |  |  |  | 2 |  | 2 |
| Marbofloxacin | 8 | 6 | 14 | 1 | 1 | 2 |  |  |  |  |  |  |  |  |  | 1 |  | 1 |
| Norfloxacin | 22 | 20 | 42 |  | 1 | 1 | 2 | 3 | 5 |  |  |  |  |  |  | 1 |  | 1 |
| Ofloxacin | 27 | 4 | 31 | 1 | 1 | 2 | 7 | 4 | 11 |  |  |  |  |  |  | 1 |  | 1 |
| Orbifloxacin | 6 |  | 6 |  |  |  | 1 | 1 | 2 |  |  |  |  |  |  |  |  |  |
| Sarafloxacin |  |  |  |  |  |  |  |  |  |  |  |  |  |  |  |  |  |  |
| **SULFONAMIDES** | **31** | **68** | **99** | **10** | **22** | **32** | **6** | **15** | **21** |  | **1** | **1** |  |  |  | **3** |  | **3** |
| Phthalylsulfathiazole |  | 6 | 6 |  |  |  |  |  |  |  |  |  |  |  |  |  |  |  |
| Sulfachlorpyridazine | 1 | 1 | 2 |  |  |  |  |  |  |  |  |  |  |  |  |  |  |  |
| Sulfadiazine | 9 | 13 | 22 | 1 | 6 | 7 | 4 | 7 | 11 |  | 1 | 1 |  |  |  | 1 |  | 1 |
| Sulfadimerazin | 1 |  | 1 |  |  |  |  |  |  |  |  |  |  |  |  |  |  |  |
| Sulfadimethoxazole | 2 | 10 | 12 | 1 |  | 1 |  |  |  |  |  |  |  |  |  |  |  |  |
| Sulfadimethoxine | 7 | 7 | 14 | 4 | 3 | 7 |  |  |  |  |  |  |  |  |  | 1 |  | 1 |
| Sulfadimidine | 2 |  | 2 | 1 |  | 1 | 2 |  | 2 |  |  |  |  |  |  |  |  |  |
| Sulfadoxine | 2 | 3 | 5 | 2 | 10 | 12 |  |  |  |  |  |  |  |  |  |  |  |  |
| Sulfafurazole |  |  |  |  |  |  |  |  |  |  |  |  |  |  |  |  |  |  |
| Sulfaguanidine |  | 7 | 7 |  | 1 | 1 |  |  |  |  |  |  |  |  |  |  |  |  |
| Sulfamerazine |  | 1 | 1 |  |  |  |  |  |  |  |  |  |  |  |  |  |  |  |
| Sulfamethazine | 4 | 6 | 10 | 1 | 1 | 2 |  | 3 | 3 |  |  |  |  |  |  | 1 |  | 1 |
| Sulfamethoxine |  | 2 | 2 |  |  |  |  |  |  |  |  |  |  |  |  |  |  |  |
| Sulfamethoxypyridazine | 1 | 3 | 4 |  |  |  |  |  |  |  |  |  |  |  |  |  |  |  |
| Sulfamonomethoxine |  | 1 | 1 |  |  |  |  |  |  |  |  |  |  |  |  |  |  |  |
| Sulfanilamide | 1 | 1 | 2 |  | 1 | 1 |  | 5 | 5 |  |  |  |  |  |  |  |  |  |
| Sulfapyridine |  |  |  |  |  |  |  |  |  |  |  |  |  |  |  |  |  |  |
| Sulfaquinoxaline | 1 | 7 | 8 |  |  |  |  |  |  |  |  |  |  |  |  |  |  |  |
| **DIAMINOPYRIMIDINES** | **7** | **4** | **11** | **2** | **2** | **4** |  |  |  |  |  |  |  |  |  |  |  |  |
| Baquiloprim |  |  |  |  |  |  |  |  |  |  |  |  |  |  |  |  |  |  |
| Trimethoprim | 7 | 3 | 10 | 2 | 2 | 4 |  |  |  |  |  |  |  |  |  |  |  |  |
| Ormetoprim | 0 | 1 | 1 |  |  |  |  |  |  |  |  |  |  |  |  |  |  |  |
| **SULFONAMIDES + DIAMINOPYRIMIDINES** | **49** | **24** | **73** | **3** | **7** | **10** | **1** | **1** | **2** |  | **1** | **1** |  |  |  | **2** |  | **2** |
| Sulfadimethoxine/Ormetoprim | 1 | 3 | 4 |  |  |  |  |  |  |  |  |  |  |  |  |  |  |  |
| Sulfonamide/Trimethoprim | 48 | 21 | 69 | 3 | 7 | 10 | 1 | 1 | 2 |  | 1 | 1 |  |  |  | 2 |  | 2 |
| **TETRACYCLINES** | **116** | **69** | **185** | **16** | **25** | **41** | **10** | **13** | **23** |  | **1** | **1** |  | **2** | **2** | **3** |  | **3** |
| Chlortetracycline | 7 | 7 | 14 |  | 2 | 2 |  | 2 | 2 |  |  |  |  | 1 | 1 |  |  |  |
| Doxycycline | 58 | 36 | 94 | 1 | 6 | 7 | 1 | 1 | 2 |  |  |  |  |  |  | 2 |  | 2 |
| Oxytetracycline | 15 | 12 | 27 | 15 | 15 | 30 | 8 | 7 | 15 |  | 1 | 1 |  | 1 | 1 | 1 |  | 1 |
| Tetracycline | 36 | 14 | 50 |  | 2 | 2 | 1 | 3 | 4 |  |  |  |  |  |  |  |  |  |

E.- English; S.- Spanish; T.- Total

**Table S2 (continuation)**: Number of websites where the individual antibiotics can be purchased according to the administration route, classified following the OIE list of antimicrobial agents of veterinary importance (22), in the searches in English and in Spanish

|  | **Oral** | | | **Parenteral** | | | **Topic** | | | **Intramammary** | | | **Intrauterin** | | | **Unknown** | | |
| --- | --- | --- | --- | --- | --- | --- | --- | --- | --- | --- | --- | --- | --- | --- | --- | --- | --- | --- |
|  | **E** | **S** | **T** | **E** | **S** | **T** | **E** | **S** | **T** | **E** | **S** | **T** | **E** | **S** | **T** | **E** | **S** | **T** |
| **HIGHLY IMPORTANT** | | | | | | | | | | | | | | | | | | |
| **ANSAMYCIN – RIFAMYCINS** | **18** | **3** | **21** |  |  |  |  | **2** | **2** |  | **2** | **2** |  | **2** | **2** |  |  |  |
| Ansamycin |  |  |  |  |  |  |  |  |  |  |  |  |  |  |  |  |  |  |
| Rifampicin | 7 | 0 | 7 |  |  |  |  |  |  |  |  |  |  |  |  |  |  |  |
| Rifaximin | 11 | 3 | 14 |  |  |  |  | 2 | 2 |  | 2 | 2 |  | 2 | 2 |  |  |  |
| **CEPHALOSPORINS** | **76** | **37** | **113** | **3** | **9** | **12** |  | **1** | **1** | **2** | **6** | **8** |  | **2** | **2** |  |  |  |
| Cefacetrile |  |  |  |  |  |  |  |  |  |  | 2 | 2 |  |  |  |  |  |  |
| Cefalexin | 51 | 27 | 78 |  | 7 | 7 |  | 1 | 1 |  | 2 | 2 |  | 1 | 1 |  |  |  |
| Cefalotin |  |  |  |  | 1 | 1 |  |  |  |  |  |  |  |  |  |  |  |  |
| Cefapyrin |  |  |  |  |  |  |  |  |  | 1 | 1 | 2 |  | 1 | 1 |  |  |  |
| Cefazolin | 2 |  | 2 | 2 | 1 | 3 |  |  |  |  |  |  |  |  |  |  |  |  |
| Cefalonium | 1 |  | 1 |  |  |  |  |  |  |  | 1 | 1 |  |  |  |  |  |  |
| Cefuroxime | 22 | 10 | 32 | 1 |  | 1 |  |  |  | 1 |  | 1 |  |  |  |  |  |  |
| **IONOPHORES** |  | **1** | **1** |  |  |  |  |  |  |  |  |  |  |  |  |  |  |  |
| Lasalocid |  |  |  |  |  |  |  |  |  |  |  |  |  |  |  |  |  |  |
| Maduramycin |  |  |  |  |  |  |  |  |  |  |  |  |  |  |  |  |  |  |
| Monensin |  |  |  |  |  |  |  |  |  |  |  |  |  |  |  |  |  |  |
| Narasin |  |  |  |  |  |  |  |  |  |  |  |  |  |  |  |  |  |  |
| Salinomycin |  | 1 | 1 |  |  |  |  |  |  |  |  |  |  |  |  |  |  |  |
| Semduramicin |  |  |  |  |  |  |  |  |  |  |  |  |  |  |  |  |  |  |
| **LINCOSAMIDES** | **22** | **13** | **35** | **8** | **8** | **16** | **1** |  | **1** | **1** | **2** | **3** |  |  |  | **1** |  | **1** |
| Pirlimycin |  |  |  |  |  |  |  |  |  | 1 |  | 1 |  |  |  |  |  |  |
| Lincomycin | 22 | 13 | 35 | 8 | 8 | 16 | 1 |  | 1 |  | 2 | 2 |  |  |  | 1 |  | 1 |
| **PHOSPHONIC ACID** | **1** | **3** | **4** |  |  |  |  |  |  |  |  |  |  |  |  |  |  |  |
| Phosphonic Acid |  |  |  |  |  |  |  |  |  |  |  |  |  |  |  |  |  |  |
| Fosfomycin | 1 | 3 | 4 |  |  |  |  |  |  |  |  |  |  |  |  |  |  |  |
| **PLEUROMUTILINS** | **1** | **4** | **5** | **1** | **2** | **3** |  |  |  |  |  |  |  |  |  | **2** |  | **2** |
| Tiamulin | 1 | 4 | 5 | 1 | 2 | 3 |  |  |  |  |  |  |  |  |  | 2 |  | 2 |
| Valnemulin |  |  |  |  |  |  |  |  |  |  |  |  |  |  |  |  |  |  |
| **POLYPEPTIDES** | **5** | **3** | **8** | **3** | **4** | **7** | **33** | **26** | **59** |  | **1** | **1** |  |  |  | **3** |  | **3** |
| Enramycin |  |  |  |  |  |  |  |  |  |  |  |  |  |  |  |  |  |  |
| Gramicidin |  |  |  |  |  |  | 1 | 3 | 4 |  |  |  |  |  |  |  |  |  |
| Bacitracin | 3 | 1 | 4 | 1 | 2 | 3 | 13 | 10 | 23 |  | 1 | 1 |  |  |  | 1 |  | 1 |
| Colistin |  | 2 | 2 | 1 | 2 | 3 | 0 | 1 | 1 |  |  |  |  |  |  | 1 |  | 1 |
| Polymixin | 2 |  | 2 | 1 |  | 1 | 19 | 12 | 31 |  |  |  |  |  |  | 1 |  | 1 |
| **QUINOLONES** | **7** | **3** | **10** |  |  |  |  |  |  |  |  |  |  |  |  |  |  |  |
| Flumequin | 1 |  | 1 |  |  |  |  |  |  |  |  |  |  |  |  |  |  |  |
| Miloxacin |  |  |  |  |  |  |  |  |  |  |  |  |  |  |  |  |  |  |
| Nalidixic acid | 6 | 3 | 9 |  |  |  |  |  |  |  |  |  |  |  |  |  |  |  |
| Oxolinic acid |  |  |  |  |  |  |  |  |  |  |  |  |  |  |  |  |  |  |

E.- English; S.- Spanish; T.- Total

**Table S2 (continuation)**: Number of websites where the individual antibiotics can be purchased according to the administration route, classified following the OIE list of antimicrobial agents of veterinary importance (22), in the searches in English and in Spanish

|  | **Oral** | | | **Parenteral** | | | **Topic** | | | **Intramammary** | | | **Intrauterin** | | | **Unknown** | | |
| --- | --- | --- | --- | --- | --- | --- | --- | --- | --- | --- | --- | --- | --- | --- | --- | --- | --- | --- |
|  | **E** | **S** | **T** | **E** | **S** | **T** | **E** | **S** | **T** | **E** | **S** | **T** | **E** | **S** | **T** | **E** | **S** | **T** |
| **IMPORTANT** | | | | | | | | | | | | | | | | | | |
| **AMINOCOUMARIN** |  |  |  |  |  |  |  |  |  |  |  |  |  |  |  |  |  |  |
| Aminocoumarin |  |  |  |  |  |  |  |  |  |  |  |  |  |  |  |  |  |  |
| Novobiocin |  |  |  |  |  |  |  |  |  |  |  |  |  |  |  |  |  |  |
| **ARSENICAL** |  |  |  |  |  |  |  |  |  |  |  |  |  |  |  |  |  |  |
| Roxarsone |  |  |  |  |  |  |  |  |  |  |  |  |  |  |  |  |  |  |
| Nitarsone |  |  |  |  |  |  |  |  |  |  |  |  |  |  |  |  |  |  |
| **BICYCLOMYCIN** |  |  |  |  |  |  |  |  |  |  |  |  |  |  |  |  |  |  |
| Bicyclomycin |  |  |  |  |  |  |  |  |  |  |  |  |  |  |  |  |  |  |
| Bicozamycin |  |  |  |  |  |  |  |  |  |  |  |  |  |  |  |  |  |  |
| **FUSIDIC ACID** | **1** | **1** | **2** |  |  |  | **6** | **1** | **7** |  |  |  |  |  |  |  |  |  |
| Fusidic acid | 1 | 1 | 2 |  |  |  | 6 | 1 | 7 |  |  |  |  |  |  |  |  |  |
| **ORTHOSOMYCINS** |  |  |  |  |  |  |  |  |  |  |  |  |  |  |  |  |  |  |
| Avilamycin |  |  |  |  |  |  |  |  |  |  |  |  |  |  |  |  |  |  |
| **QUINOXALINES** |  |  |  |  |  |  |  |  |  |  |  |  |  |  |  |  |  |  |
| Carbadox |  |  |  |  |  |  |  |  |  |  |  |  |  |  |  |  |  |  |
| Olaquindox |  |  |  |  |  |  |  |  |  |  |  |  |  |  |  |  |  |  |
| **STREPTOGRAMINS** |  |  |  |  |  |  |  |  |  |  |  |  |  |  |  |  |  |  |
| Virginiamycin |  |  |  |  |  |  |  |  |  |  |  |  |  |  |  |  |  |  |
| **THIOSTREPTON** |  |  |  |  |  |  | **2** | **2** | **4** |  |  |  |  |  |  |  |  |  |
| Thiostrepton |  |  |  |  |  |  | 2 | 2 | 4 |  |  |  |  |  |  |  |  |  |
| Nosiheptide |  |  |  |  |  |  |  |  |  |  |  |  |  |  |  |  |  |  |

E.- English; S.- Spanish; T.- Total

**Table S3**: Number of websites where the individual antibiotics can be purchased according to the species they are intended to, classified following the OIE list of antimicrobial agents of veterinary importance (22), in the searches in English and in Spanish

|  | **All species** | | | **Bovine** | | | **Ovine** | | | **Caprine** | | | **Pigs** | | | **Horses** | | | **Poultry** | | | **Dogs** | | | **Cats and dogs** | | | **Fish** | | | **Humans** | | | **Animals and humans** | | | **Others** | | |
| --- | --- | --- | --- | --- | --- | --- | --- | --- | --- | --- | --- | --- | --- | --- | --- | --- | --- | --- | --- | --- | --- | --- | --- | --- | --- | --- | --- | --- | --- | --- | --- | --- | --- | --- | --- | --- | --- | --- | --- |
|  | **E** | **S** | **T** | **E** | **S** | **T** | **E** | **S** | **T** | **E** | **S** | **T** | **E** | **S** | **T** | **E** | **S** | **T** | **E** | **S** | **T** | **E** | **S** | **T** | **E** | **S** | **T** | **E** | **S** | **T** | **E** | **S** | **T** | **E** | **S** | **T** | **E** | **S** | **T** |
| **CRITICALLY IMPORTANT** | | | | | | | | | | | | | | | | | | | | | | | | | | | | | | | | | | | | | | | |
| **AMINOGLYCOSIDES** | **6** | **25** | **31** | **11** | **54** | **65** | **6** | **34** | **40** | **8** | **30** | **38** | **10** | **37** | **47** | **14** | **38** | **52** | **8** | **14** | **22** | **6** | **12** | **18** | **25** | **56** | **81** |  |  |  | **26** | **13** | **39** | **7** | **2** | **9** | **11** | **7** | **18** |
| Aminocyclitol |  |  |  |  |  |  |  |  |  |  |  |  |  |  |  |  |  |  |  |  |  |  |  |  |  |  |  |  |  |  |  |  |  |  |  |  |  |  |  |
| Amikacin |  |  |  | 1 |  | 1 |  |  |  |  |  |  |  |  |  | 2 | 1 | 3 | 1 |  | 1 |  |  |  |  | 1 | 1 |  |  |  | 3 | 3 | 6 | 1 |  | 1 |  |  |  |
| Apramycin |  |  |  |  |  |  |  |  |  |  |  |  |  |  |  |  |  |  |  |  |  |  |  |  |  |  |  |  |  |  |  |  |  |  |  |  |  |  |  |
| Dihydrostreptomycin |  | 13 | 13 | 2 | 8 | 10 |  | 7 | 7 | 1 | 8 | 9 | 1 | 8 | 9 | 1 | 3 | 4 |  |  |  |  | 9 | 9 |  |  |  |  |  |  |  |  |  |  |  |  | 7 |  | 7 |
| Fortimycin |  |  |  |  |  |  |  |  |  |  |  |  |  |  |  |  |  |  |  |  |  |  |  |  |  |  |  |  |  |  |  |  |  |  |  |  |  |  |  |
| Framycetin |  |  |  |  | 1 | 1 |  |  |  |  |  |  |  |  |  |  |  |  |  |  |  |  |  |  | 1 | 2 | 3 |  |  |  |  |  |  |  |  |  |  |  |  |
| Gentamicin | 1 | 7 | 8 |  | 15 | 15 |  | 11 | 11 |  | 7 | 7 | 1 | 13 | 14 | 5 | 13 | 18 |  | 6 | 6 | 3 | 1 | 4 | 6 | 19 | 25 |  |  |  | 7 | 3 | 10 | 1 | 1 | 2 |  | 4 | 4 |
| Kanamycin |  |  |  | 1 | 3 | 4 |  | 1 | 1 | 1 |  | 1 | 1 |  | 1 | 1 | 2 | 3 |  | 1 | 1 |  |  |  |  |  |  |  |  |  | 1 |  | 1 | 1 |  | 1 |  |  |  |
| Neomycin | 5 | 2 | 7 | 7 | 13 | 20 | 6 | 4 | 10 | 6 | 5 | 11 | 5 | 6 | 11 | 4 | 9 | 13 | 3 | 5 | 8 | 2 | 1 | 3 | 14 | 19 | 33 |  |  |  | 8 | 3 | 11 | 1 | 1 | 2 | 2 | 3 | 5 |
| Paromomycin |  |  |  |  |  |  |  |  |  |  |  |  |  |  |  |  |  |  |  |  |  |  |  |  |  |  |  |  |  |  |  |  |  |  |  |  |  |  |  |
| Spectinomycin |  |  |  |  | 2 | 2 |  | 2 | 2 |  | 2 | 2 | 1 | 3 | 4 | 1 |  | 1 | 3 | 1 | 4 |  |  |  | 1 | 2 | 3 |  |  |  |  |  |  | 1 |  | 1 | 1 |  | 1 |
| Streptomycin |  | 3 | 3 |  | 11 | 11 |  | 8 | 8 |  | 7 | 7 | 1 | 6 | 7 |  | 9 | 9 | 1 | 1 | 2 |  | 1 | 1 |  | 5 | 5 |  |  |  |  |  |  | 1 |  | 1 | 1 |  | 1 |
| Tobramycin |  |  |  |  | 1 | 1 |  | 1 | 1 |  | 1 | 1 |  | 1 | 1 |  | 1 | 1 |  |  |  | 1 |  | 1 | 3 | 8 | 11 |  |  |  | 7 | 4 | 11 | 1 |  | 1 |  |  |  |
| **AMPHENICOLS** | **1** | **2** | **3** | **4** | **12** | **16** |  | **2** | **2** |  |  |  |  | **12** | **12** |  | **1** | **1** |  | **4** | **4** |  | **1** | **1** | **2** | **1** | **3** |  |  |  |  |  |  |  |  |  |  | **2** | **2** |
| Florphenicol | 1 | 2 | 3 | 4 | 11 | 15 |  | 1 | 1 |  |  |  |  | 11 | 11 |  |  |  |  | 3 | 3 |  | 1 | 1 | 2 |  | 2 |  |  |  |  |  |  |  |  |  |  |  |  |
| Thiamphenicol |  |  |  |  | 1 | 1 |  | 1 | 1 |  |  |  |  | 1 | 1 |  | 1 | 1 |  | 1 | 1 |  |  |  |  | 1 | 1 |  |  |  |  |  |  |  |  |  |  | 2 | 2 |
| **CEPHALOSPORINS** | **1** | **2** | **3** | **6** | **18** | **24** | **1** |  | **1** | **1** |  | **1** | **2** | **17** | **19** | **3** | **5** | **8** | **1** | **5** | **6** |  | **1** | **1** | **1** | **2** | **3** |  |  |  | **5** | **3** | **8** |  |  |  |  |  |  |
| Cefoperazone |  |  |  |  |  |  |  |  |  |  |  |  |  |  |  |  |  |  |  |  |  |  |  |  |  |  |  |  |  |  | 1 |  | 1 |  |  |  |  |  |  |
| Ceftiofur | 1 | 1 | 2 | 5 | 15 | 20 | 1 |  | 1 | 1 |  | 1 | 2 | 14 | 16 | 3 | 5 | 8 | 1 | 5 | 6 |  |  |  | 1 | 2 | 3 |  |  |  |  |  |  |  |  |  |  |  |  |
| Ceftriaxone |  |  |  | 1 |  | 1 |  |  |  |  |  |  |  |  |  |  |  |  |  |  |  |  |  |  |  |  |  |  |  |  | 4 | 3 | 7 |  |  |  |  |  |  |
| Cefquinoma |  | 1 | 1 |  | 3 | 3 |  |  |  |  |  |  |  | 3 | 3 |  |  |  |  |  |  |  | 1 | 1 |  |  |  |  |  |  |  |  |  |  |  |  |  |  |  |
| **MACROLIDES** |  | **7** | **7** | **16** | **31** | **47** | **2** | **7** | **9** | **1** | **4** | **5** | **13** | **16** | **29** | **2** | **0** | **2** | **2** | **11** | **13** | **1** | **2** | **3** | **12** | **23** | **35** | **6** | **0** | **6** | **35** | **8** | **43** | **3** |  | **3** | **6** | **4** | **10** |
| Carbomycin |  |  |  |  |  |  |  |  |  |  |  |  |  |  |  |  |  |  |  |  |  |  |  |  |  |  |  |  |  |  |  |  |  |  |  |  |  |  |  |
| Erythromycin |  | 4 | 4 |  | 2 | 2 |  |  |  |  |  |  |  | 1 | 1 | 1 |  | 1 |  |  |  |  |  |  | 6 | 3 | 9 | 5 |  | 5 | 33 | 8 | 41 | 1 |  | 1 | 1 |  | 1 |
| Gamithromycin |  |  |  | 1 | 1 | 2 | 1 |  | 1 |  |  |  | 1 | 1 | 2 |  |  |  |  |  |  |  |  |  |  |  |  |  |  |  |  |  |  |  |  |  |  |  |  |
| Josamycin |  |  |  |  |  |  |  |  |  |  |  |  |  |  |  |  |  |  |  |  |  |  |  |  |  |  |  |  |  |  |  |  |  |  |  |  |  |  |  |
| Kitasamycin |  |  |  |  |  |  |  |  |  |  |  |  |  |  |  |  |  |  |  | 1 | 1 |  |  |  |  |  |  |  |  |  |  |  |  |  |  |  |  |  |  |
| Mirosamycin |  |  |  |  |  |  |  |  |  |  |  |  |  |  |  |  |  |  |  |  |  |  |  |  |  |  |  |  |  |  |  |  |  |  |  |  |  |  |  |
| Oleandomycin |  |  |  |  |  |  |  |  |  |  |  |  |  |  |  |  |  |  |  |  |  |  |  |  |  |  |  |  |  |  | 2 |  | 2 | 1 |  | 1 |  |  |  |
| Sedecamycin |  |  |  |  |  |  |  |  |  |  |  |  |  |  |  |  |  |  |  |  |  |  |  |  |  |  |  |  |  |  |  |  |  |  |  |  |  |  |  |
| Spiramycin |  | 1 | 1 |  | 8 | 8 |  |  |  |  |  |  |  | 1 | 1 |  |  |  |  | 1 | 1 | 0 | 1 | 1 | 0 | 16 | 16 |  |  |  |  |  |  |  |  |  | 2 |  | 2 |
| Terdecamycin |  |  |  |  |  |  |  |  |  |  |  |  |  |  |  |  |  |  |  |  |  |  |  |  |  |  |  |  |  |  |  |  |  |  |  |  |  |  |  |
| Tildipirosin |  |  |  | 3 |  | 3 |  |  |  |  |  |  |  |  |  |  |  |  |  |  |  |  |  |  |  |  |  |  |  |  |  |  |  |  |  |  |  |  |  |
| Tilmicosin |  |  |  |  | 8 | 8 |  | 2 | 2 |  |  |  | 2 | 1 | 3 |  |  |  |  | 1 | 1 |  |  |  |  |  |  |  |  |  |  |  |  |  |  |  |  |  |  |
| Tulathromycin |  |  |  | 4 | 4 | 8 |  |  |  |  |  |  | 3 | 4 | 7 |  |  |  |  |  |  |  |  |  |  |  |  |  |  |  |  |  |  |  |  |  |  |  |  |
| Tylosin |  | 2 | 2 | 8 | 8 | 16 | 1 | 5 | 6 | 1 | 4 | 5 | 7 | 7 | 14 | 1 |  | 1 | 2 | 8 | 10 | 1 | 1 | 2 | 4 | 4 | 8 | 1 |  | 1 |  |  |  | 1 |  | 1 | 3 | 4 | 7 |
| Tylvalosin |  |  |  |  |  |  |  |  |  |  |  |  |  |  |  |  |  |  |  |  |  |  |  |  | 2 |  | 2 |  |  |  |  |  |  |  |  |  |  |  |  |

**Table S3 (continuation)**: Number of websites where the individual antibiotics can be purchased according to the species they are intended to, classified following the OIE list of antimicrobial agents of veterinary importance (22), in the searches in English and in Spanish

|  | **All species** | | | **Bovine** | | | **Ovine** | | | **Caprine** | | | **Pigs** | | | **Horses** | | | **Poultry** | | | **Dogs** | | | **Cats and dogs** | | | **Fish** | | | **Humans** | | | **Animals and humans** | | | **Others** | | |
| --- | --- | --- | --- | --- | --- | --- | --- | --- | --- | --- | --- | --- | --- | --- | --- | --- | --- | --- | --- | --- | --- | --- | --- | --- | --- | --- | --- | --- | --- | --- | --- | --- | --- | --- | --- | --- | --- | --- | --- |
|  | **E** | **S** | **T** | **E** | **S** | **T** | **E** | **S** | **T** | **E** | **S** | **T** | **E** | **S** | **T** | **E** | **S** | **T** | **E** | **S** | **T** | **E** | **S** | **T** | **E** | **S** | **T** | **E** | **S** | **T** | **E** | **S** | **T** | **E** | **S** | **T** | **E** | **S** | **T** |
| **CRITICALLY IMPORTANT** | | | | | | | | | | | | | | | | | | | | | | | | | | | | | | | | | | | | | | | |
| **PENICILLINS** | **3** | **22** | **25** | **20** | **73** | **93** | **10** | **41** | **51** | **3** | **26** | **29** | **22** | **43** | **65** | **12** | **32** | **44** | **1** | **18** | **19** | **4** | **3** | **7** | **24** | **69** | **93** | **24** |  | **24** | **127** | **39** | **166** | **2** | **3** | **5** | **5** | **1** | **6** |
| **Natural Penicillins** |  |  |  |  |  |  |  |  |  |  |  |  |  |  |  |  |  |  |  |  |  |  |  |  |  |  |  |  |  |  |  |  |  |  |  |  |  |  |  |
| Benethamine penicillin |  |  |  |  | 1 | 1 |  |  |  |  |  |  |  |  |  |  |  |  |  |  |  |  |  |  |  |  |  | 1 |  | 1 |  |  |  |  |  |  |  |  |  |
| Benzylpenicillin |  | 1 | 1 |  | 9 | 9 |  | 9 | 9 |  | 7 | 7 |  | 10 | 10 |  | 10 | 10 |  | 2 | 2 |  |  |  |  | 7 | 7 | 4 |  | 4 | 2 | 2 | 4 |  |  |  | 1 |  | 1 |
| Penethamate |  |  |  |  | 3 | 3 |  | 1 | 1 |  | 1 | 1 |  | 1 | 1 |  |  |  |  |  |  |  |  |  |  |  |  |  |  |  |  |  |  |  |  |  |  |  |  |
| Bencilpenicillin procaine |  | 4 | 4 | 11 | 14 | 25 | 10 | 9 | 19 | 3 | 6 | 9 | 10 | 11 | 21 | 9 | 7 | 16 |  | 1 | 1 |  |  |  | 1 | 7 | 8 |  |  |  | 2 |  | 2 |  |  |  |  |  |  |
| Benzatine penicillin |  | 5 | 5 | 3 | 13 | 16 |  | 5 | 5 |  | 3 | 3 |  | 5 | 5 |  | 7 | 7 |  | 1 | 1 |  | 1 | 1 | 1 | 5 | 6 |  |  |  | 2 | 1 | 3 |  |  |  |  |  |  |
| **Amdinopenicillins** |  |  |  |  |  |  |  |  |  |  |  |  |  |  |  |  |  |  |  |  |  |  |  |  |  |  |  |  |  |  |  |  |  |  |  |  |  |  |  |
| Mecillinam |  |  |  |  |  |  |  |  |  |  |  |  |  |  |  |  |  |  |  |  |  |  |  |  |  |  |  |  |  |  |  |  |  |  |  |  |  |  |  |
| **Aminopenicillins** |  |  |  |  |  |  |  |  |  |  |  |  |  |  |  |  |  |  |  |  |  |  |  |  |  |  |  |  |  |  |  |  |  |  |  |  |  |  |  |
| Amoxicillin | 2 | 6 | 8 | 1 | 12 | 13 |  | 7 | 7 |  | 2 | 2 | 12 | 10 | 22 | 1 | 2 | 3 | 1 | 3 | 4 | 2 | 1 | 3 | 9 | 25 | 34 | 12 |  | 12 | 37 | 12 | 49 | 1 | 1 | 2 | 3 |  | 3 |
| Amoxicillin/Clavulanic Acid |  | 3 | 3 |  | 1 | 1 |  |  |  |  |  |  |  |  |  | 1 |  | 1 |  |  |  | 1 | 1 | 2 | 9 | 13 | 22 | 1 |  | 1 | 34 | 8 | 42 |  | 1 | 1 |  |  |  |
| Ampicillin |  | 2 | 2 | 2 | 10 | 12 |  | 7 | 7 |  | 4 | 4 |  | 5 | 5 | 1 | 6 | 7 |  | 2 | 2 | 1 |  | 1 | 4 | 11 | 15 | 3 |  | 3 | 30 | 10 | 40 | 1 | 1 | 2 | 1 | 1 | 2 |
| Ampicillin/Sulbactam | 1 |  | 1 |  |  |  |  |  |  |  |  |  |  |  |  |  |  |  |  |  |  |  |  |  |  |  |  |  |  |  | 3 | 1 | 4 |  |  |  |  |  |  |
| Hetacillin |  |  |  | 1 |  | 1 |  |  |  |  |  |  |  |  |  |  |  |  |  |  |  |  |  |  |  |  |  |  |  |  |  |  |  |  |  |  |  |  |  |
| **Carboxypenicillins** |  |  |  |  |  |  |  |  |  |  |  |  |  |  |  |  |  |  |  |  |  |  |  |  |  |  |  |  |  |  |  |  |  |  |  |  |  |  |  |
| Ticarcillin |  |  |  |  |  |  |  |  |  |  |  |  |  |  |  |  |  |  |  |  |  |  |  |  |  |  |  |  |  |  |  |  |  |  |  |  |  |  |  |
| Tobicillin |  |  |  |  |  |  |  |  |  |  |  |  |  |  |  |  |  |  |  |  |  |  |  |  |  |  |  |  |  |  |  |  |  |  |  |  |  |  |  |
| **Ureidopenicillin** |  |  |  |  |  |  |  |  |  |  |  |  |  |  |  |  |  |  |  |  |  |  |  |  |  |  |  |  |  |  |  |  |  |  |  |  |  |  |  |
| Aspoxicillin |  |  |  |  |  |  |  |  |  |  |  |  |  |  |  |  |  |  |  |  |  |  |  |  |  |  |  |  |  |  |  |  |  |  |  |  |  |  |  |
| **Phenoxypenicillins** |  |  |  |  |  |  |  |  |  |  |  |  |  |  |  |  |  |  |  |  |  |  |  |  |  |  |  |  |  |  |  |  |  |  |  |  |  |  |  |
| Phenoxymethylpenicillin |  |  |  |  |  |  |  |  |  |  |  |  |  | 1 | 1 |  |  |  |  | 1 | 1 |  |  |  |  |  |  | 3 |  | 3 | 3 | 1 | 4 |  |  |  |  |  |  |
| Phenethicillin |  |  |  |  |  |  |  |  |  |  |  |  |  |  |  |  |  |  |  |  |  |  |  |  |  |  |  |  |  |  |  |  |  |  |  |  |  |  |  |
| **Antistaphylococcal Penicillins** |  |  |  |  |  |  |  |  |  |  |  |  |  |  |  |  |  |  |  |  |  |  |  |  |  |  |  |  |  |  |  |  |  |  |  |  |  |  |  |
| Cloxacillin |  | 1 | 1 | 2 | 9 | 11 |  | 3 | 3 |  | 3 | 3 |  |  |  |  |  |  |  |  |  |  |  |  |  | 1 | 1 |  |  |  | 6 | 3 | 9 |  |  |  |  |  |  |
| Dicloxacillin |  |  |  |  |  |  |  |  |  |  |  |  |  |  |  |  |  |  |  |  |  |  |  |  |  |  |  |  |  |  | 3 | 1 | 4 |  |  |  |  |  |  |
| Nafcillin |  |  |  |  | 1 | 1 |  |  |  |  |  |  |  |  |  |  |  |  |  |  |  |  |  |  |  |  |  |  |  |  | 3 |  | 3 |  |  |  |  |  |  |
| Oxacillin |  |  |  |  |  |  |  |  |  |  |  |  |  |  |  |  |  |  |  |  |  |  |  |  |  |  |  |  |  |  | 2 |  | 2 |  |  |  |  |  |  |
| **FLUOROQUINOLONES** | **2** | **7** | **9** | **9** | **19** | **28** | **1** | **10** | **11** |  | **6** | **6** | **5** | **16** | **21** | **1** | **8** | **9** | **1** | **18** | **19** |  | **10** | **10** | **32** | **58** | **90** | **12** |  | **12** | **84** | **26** | **110** | **3** | **1** | **4** | **3** | **5** | **8** |
| Ciprofloxacin |  |  |  | 2 | 1 | 3 | 1 |  | 1 |  |  |  | 1 | 1 | 2 | 1 | 2 | 3 |  | 1 | 1 |  |  |  | 6 | 13 | 19 | 12 |  | 12 | 35 | 9 | 44 | 1 | 1 | 2 |  |  |  |
| Danofloxacin |  | 1 | 1 | 2 | 2 | 4 |  |  |  |  |  |  | 1 |  | 1 |  |  |  |  |  |  |  |  |  |  |  |  |  |  |  |  |  |  |  |  |  |  |  |  |
| Difloxacin |  |  |  |  |  |  |  |  |  |  |  |  |  |  |  |  |  |  |  |  |  |  |  |  |  |  |  |  |  |  | 1 | 1 | 2 |  |  |  |  |  |  |
| Enrofloxacin | 2 | 4 | 6 | 3 | 14 | 17 |  | 9 | 9 |  | 5 | 5 | 3 | 13 | 16 |  | 5 | 5 | 1 | 16 | 17 |  | 4 | 4 | 10 | 31 | 41 |  |  |  |  |  |  | 1 |  | 1 | 3 | 5 | 8 |
| Marbofloxacin |  |  |  | 1 | 1 | 2 |  |  |  |  |  |  |  | 1 | 1 |  |  |  |  |  |  |  | 3 | 3 | 8 | 3 | 11 |  |  |  |  |  |  | 1 |  | 1 |  |  |  |
| Norfloxacin |  | 2 | 2 |  | 1 | 1 |  | 1 | 1 |  | 1 | 1 |  | 1 | 1 |  | 1 | 1 |  | 1 | 1 |  | 2 | 2 |  | 9 | 9 |  |  |  | 21 | 9 | 30 |  |  |  |  |  |  |
| Ofloxacin |  |  |  | 1 |  | 1 |  |  |  |  |  |  |  |  |  |  |  |  |  |  |  |  |  |  | 2 | 2 | 4 |  |  |  | 27 | 7 | 34 |  |  |  |  |  |  |
| Orbifloxacin |  |  |  |  |  |  |  |  |  |  |  |  |  |  |  |  |  |  |  |  |  |  | 1 | 1 | 6 |  | 6 |  |  |  |  |  |  |  |  |  |  |  |  |
| Sarafloxacin |  |  |  |  |  |  |  |  |  |  |  |  |  |  |  |  |  |  |  |  |  |  |  |  |  |  |  |  |  |  |  |  |  |  |  |  |  |  |  |

**Table S3 (continuation)**: Number of websites where the individual antibiotics can be purchased according to the species they are intended to, classified following the OIE list of antimicrobial agents of veterinary importance (22), in the searches in English and in Spanish

|  | **All species** | | | **Bovine** | | | **Ovine** | | | **Caprine** | | | **Pigs** | | | **Horses** | | | **Poultry** | | | **Dogs** | | | **Cats and dogs** | | | **Fish** | | | **Humans** | | | **Animals and humans** | | | **Others** | | |  |  |  |
| --- | --- | --- | --- | --- | --- | --- | --- | --- | --- | --- | --- | --- | --- | --- | --- | --- | --- | --- | --- | --- | --- | --- | --- | --- | --- | --- | --- | --- | --- | --- | --- | --- | --- | --- | --- | --- | --- | --- | --- | --- | --- | --- |
|  | **E** | **S** | **T** | **E** | **S** | **T** | **E** | **S** | **T** | **E** | **S** | **T** | **E** | **S** | **T** | **E** | **S** | **T** | **E** | **S** | **T** | **E** | **S** | **T** | **E** | **S** | **T** | **E** | **S** | **T** | **E** | **S** | **T** | **E** | **S** | **T** | **E** | **S** | **T** |  |  |  |
| **CRITICALLY IMPORTANT** | | | | | | | | | | | | | | | | | | | | | | | | | | | | | | | | | | | | | | | |  |  |  |
| **SULFONAMIDES** | **3** | **10** | **13** | **12** | **33** | **45** | **4** | **22** | **26** | **2** | **17** | **19** | **1** | **26** | **27** | **5** | **27** | **32** | **5** | **8** | **13** | **1** | **17** | **18** | **11** | **30** | **41** | **1** | **2** | **3** | **5** | **8** | **13** | **2** | **4** | **6** | **5** | **4** | **9** |  |  |  |
| Phthalylsulfathiazole |  |  |  |  | 4 | 4 |  | 2 | 2 |  |  |  |  | 3 | 3 |  | 1 | 1 |  | 1 | 1 |  |  |  |  | 2 | 2 |  |  |  |  | 1 | 1 |  |  |  |  |  |  |  |  |  |
| Sulfachlorpyridazine |  |  |  |  | 1 | 1 |  | 1 | 1 |  |  |  |  | 1 | 1 |  |  |  |  |  |  |  |  |  |  |  |  |  |  |  |  |  |  |  |  |  | 1 |  | 1 |  |  |  |
| Sulfadiazine | 1 | 1 | 2 | 1 | 8 | 9 | 1 | 5 | 6 |  | 4 | 4 |  | 6 | 6 | 5 | 5 | 10 | 1 | 2 | 3 |  | 2 | 2 | 3 | 7 | 10 |  |  |  | 3 | 2 | 5 | 1 |  | 1 |  | 1 | 1 |  |  |  |
| Sulfadimerazin |  |  |  |  | 1 | 1 |  |  |  |  |  |  |  |  |  |  |  |  | 1 |  | 1 |  |  |  |  |  |  |  |  |  |  |  |  |  |  |  |  |  |  |  |  |  |
| Sulfadimethoxazole |  |  |  |  | 1 | 1 |  | 1 | 1 |  | 1 | 1 |  | 2 | 2 |  |  |  |  | 2 | 2 |  |  |  | 2 | 4 | 6 | 1 |  | 1 |  | 4 | 4 |  |  |  |  |  |  | 0 | 0 | 0 |
| Sulfadimethoxine | 1 | 3 | 4 | 6 | 2 | 8 | 1 |  | 1 | 1 | 3 | 4 |  | 3 | 3 |  |  |  | 1 |  | 1 | 1 | 9 | 10 | 6 |  | 6 |  |  |  |  |  |  | 1 | 1 | 2 |  |  |  |  |  |  |
| Sulfadimidine | 1 |  | 1 |  |  |  |  |  |  |  |  |  |  |  |  |  |  |  | 2 |  | 2 |  |  |  |  |  |  |  |  |  |  |  |  |  |  |  | 2 |  | 2 |  |  |  |
| Sulfadoxine |  | 3 | 3 | 2 | 8 | 10 | 1 | 7 | 8 |  | 3 | 3 |  | 7 | 7 |  | 8 | 8 |  | 1 | 1 |  | 1 | 1 |  | 4 | 4 |  |  |  | 1 |  | 1 |  |  |  |  | 1 | 1 |  |  |  |
| Sulfafurazole |  |  |  |  |  |  |  |  |  |  |  |  |  |  |  |  |  |  |  |  |  |  |  |  |  |  |  |  |  |  |  |  |  |  |  |  |  |  |  |  |  |  |
| Sulfaguanidine |  | 1 | 1 |  | 1 | 1 |  | 1 | 1 |  | 1 | 1 |  | 1 | 1 |  | 4 | 4 |  |  |  |  | 4 | 4 |  |  |  |  | 1 | 1 |  | 1 | 1 |  |  |  |  |  |  |  |  |  |
| Sulfamerazine |  |  |  |  | 1 | 1 |  |  |  |  |  |  |  |  |  |  |  |  |  |  |  |  |  |  |  |  |  |  |  |  |  |  |  |  |  |  |  |  |  |  |  |  |
| Sulfamethazine |  |  |  | 3 | 4 | 7 | 1 | 3 | 4 | 1 | 1 | 2 | 1 | 3 | 4 |  | 4 | 4 |  | 1 | 1 |  | 1 | 1 |  | 6 | 6 |  |  |  |  |  |  |  |  |  |  |  |  |  |  |  |
| Sulfamethoxine |  |  |  |  |  |  |  |  |  |  |  |  |  |  |  |  |  |  |  |  |  |  |  |  |  | 2 | 2 |  |  |  |  |  |  |  |  |  |  |  |  |  |  |  |
| Sulfamethoxypyridazine |  | 1 | 1 |  |  |  |  |  |  |  | 1 | 1 |  |  |  |  | 2 | 2 |  |  |  |  |  |  |  |  |  |  |  |  |  |  |  |  |  |  | 1 |  | 1 |  |  |  |
| Sulfamonomethoxine |  | 1 | 1 |  | 1 | 1 |  |  |  |  | 1 | 1 |  |  |  |  | 1 | 1 |  |  |  |  |  |  |  |  |  |  |  |  |  |  |  |  | 3 | 3 |  |  |  |  |  |  |
| Sulfanilamide |  |  |  |  | 1 | 1 |  | 1 | 1 |  | 1 | 1 |  |  |  |  | 2 | 2 |  | 1 | 1 |  |  |  |  | 4 | 4 |  | 1 | 1 | 1 |  | 1 |  |  |  |  |  |  |  |  |  |
| Sulfapyridine |  |  |  |  |  |  |  |  |  |  |  |  |  |  |  |  |  |  |  |  |  |  |  |  |  |  |  |  |  |  |  |  |  |  |  |  |  |  |  |  |  |  |
| Sulfaquinoxaline |  |  |  |  |  |  |  | 1 | 1 |  | 1 | 1 |  |  |  |  |  |  |  | 5 | 5 |  |  |  |  | 1 | 1 |  |  |  |  |  |  |  |  |  | 1 | 2 | 3 |  |  |  |
| **DIAMINOPYRIMIDINES** |  |  |  |  | **4** | **4** |  |  |  |  |  |  |  |  |  | **2** | **5** | **7** |  |  |  |  |  |  | **3** | **7** | **10** | **2** |  | **2** | **12** | **1** | **13** |  |  |  |  |  |  |  |  |  |
| Baquiloprim |  |  |  |  |  |  |  |  |  |  |  |  |  |  |  |  |  |  |  |  |  |  |  |  |  |  |  |  |  |  |  |  |  |  |  |  |  |  |  |  |  |  |
| Trimethoprim |  |  |  |  | 4 | 4 |  |  |  |  |  |  |  |  |  | 2 | 5 | 7 |  |  |  |  |  |  | 3 | 7 | 10 | 2 |  | 2 | 12 | 1 | 13 |  |  |  |  |  |  |  |  |  |
| Ormetoprim |  |  |  |  |  |  |  |  |  |  |  |  |  |  |  |  |  |  |  |  |  |  |  |  |  | 1 | 1 |  |  |  |  |  |  |  |  |  |  |  |  |  |  |  |
| **SULFONAMIDES + DIAMINOPYRIMIDINES** | **1** | **4** | **5** | **1** | **9** | **10** | **2** | **9** | **11** |  | **5** | **5** |  | **11** | **11** | **4** | **8** | **12** |  | **6** | **6** |  |  |  | **6** | **9** | **15** | **7** |  | **7** | **21** | **5** | **26** | **1** |  | **1** | **5** | **5** | **10** |  |  |  |
| Sulfadimethoxine/Ormetoprim |  |  |  |  |  |  |  |  |  |  |  |  |  |  |  |  | 1 | 1 |  |  |  |  |  |  |  | 3 | 3 |  |  |  |  |  |  |  |  |  |  |  |  |  |  |  |
| Sulfonamide/Trimethoprim | 1 | 4 | 5 | 1 | 9 | 10 | 2 | 9 | 11 |  | 5 | 5 |  | 11 | 11 | 4 | 7 | 11 |  | 6 | 6 |  |  |  | 6 | 6 | 12 | 7 |  | 7 | 21 | 5 | 26 | 1 |  | 1 | 5 | 5 | 10 |  |  |  |
| **TETRACYCLINES** | **3** | **8** | **11** | **21** | **32** | **53** | **7** | **24** | **31** | **4** | **16** | **20** | **16** | **28** | **44** | **5** | **17** | **22** | **13** | **21** | **34** | **3** |  | **3** | **17** | **41** | **58** | **11** |  | **11** | **60** | **18** | **78** | **2** | **1** | **3** | **19** | **10** | **29** |  |  |  |
| Chlortetracycline |  |  |  | 1 | 5 | 6 |  | 3 | 3 |  | 3 | 3 | 1 | 5 | 6 |  | 3 | 3 | 4 | 6 | 10 |  |  |  |  | 3 | 3 |  |  |  |  |  |  |  |  |  | 3 |  | 3 |  |  |  |
| Doxycycline | 1 | 2 | 3 | 3 | 4 | 7 | 1 | 3 | 4 | 1 | 3 | 4 | 1 | 6 | 7 | 1 |  | 1 | 2 | 5 | 7 | 3 |  | 3 | 8 | 24 | 32 | 6 |  | 6 | 30 | 10 | 40 | 1 | 1 | 2 | 12 | 2 | 14 |  |  |  |
| Oxytetracycline | 1 | 5 | 6 | 15 | 18 | 33 | 6 | 13 | 19 | 3 | 9 | 12 | 12 | 12 | 24 | 4 | 11 | 15 | 4 | 8 | 12 |  |  |  | 6 | 10 | 16 | 2 |  | 2 | 4 | 1 | 5 | 1 |  | 1 | 3 | 6 | 9 |  |  |  |
| Tetracycline | 1 | 1 | 2 | 2 | 5 | 7 |  | 5 | 5 |  | 1 | 1 | 2 | 5 | 7 |  | 3 | 3 | 3 | 2 | 5 |  |  |  | 3 | 4 | 7 | 3 |  | 3 | 26 | 7 | 33 |  |  |  | 1 | 2 | 3 |  |  |  |

**Table S3 (continuation)**: Number of websites where the individual antibiotics can be purchased according to the species they are intended to, classified following the OIE list of antimicrobial agents of veterinary importance (22), in the searches in English and in Spanish

|  | **All species** | | | **Bovine** | | | **Ovine** | | | **Caprine** | | | **Pigs** | | | **Horses** | | | **Poultry** | | | **Dogs** | | | **Cats and dogs** | | | **Fish** | | | **Humans** | | | **Animals and humans** | | | **Others** | | |
| --- | --- | --- | --- | --- | --- | --- | --- | --- | --- | --- | --- | --- | --- | --- | --- | --- | --- | --- | --- | --- | --- | --- | --- | --- | --- | --- | --- | --- | --- | --- | --- | --- | --- | --- | --- | --- | --- | --- | --- |
|  | **E** | **S** | **T** | **E** | **S** | **T** | **E** | **S** | **T** | **E** | **S** | **T** | **E** | **S** | **T** | **E** | **S** | **T** | **E** | **S** | **T** | **E** | **S** | **T** | **E** | **S** | **T** | **E** | **S** | **T** | **E** | **S** | **T** | **E** | **S** | **T** | **E** | **S** | **T** |
| **HIGHLY IMPORTANT** | | | | | | | | | | | | | | | | | | | | | | | | | | | | | | | | | | | | | | | |
| **CEPHALOSPORINS** |  | **1** | **1** | **2** | **11** | **13** | **1** | **2** | **3** |  | **1** | **1** |  | **2** | **2** | **2** | **4** | **6** |  |  |  | **1** |  | **1** | **13** | **20** | **33** | **15** |  | **15** | **64** | **19** | **83** |  |  |  |  |  |  |
| Cefacetrile |  |  |  |  | 2 | 2 |  |  |  |  |  |  |  |  |  |  |  |  |  |  |  |  |  |  |  |  |  |  |  |  |  |  |  |  |  |  |  |  |  |
| Cefalexin |  | 1 | 1 |  | 7 | 7 |  | 2 | 2 |  | 1 | 1 |  | 2 | 2 |  | 4 | 4 |  |  |  | 1 |  | 1 | 8 | 19 | 27 | 12 |  | 12 | 30 | 8 | 38 |  |  |  |  |  |  |
| Cefalotin |  |  |  |  |  |  |  |  |  |  |  |  |  |  |  |  |  |  |  |  |  |  |  |  |  | 1 | 1 |  |  |  |  |  |  |  |  |  |  |  |  |
| Cefapyrin |  |  |  | 1 | 1 | 2 |  |  |  |  |  |  |  |  |  |  |  |  |  |  |  |  |  |  |  |  |  |  |  |  |  |  |  |  |  |  |  |  |  |
| Cefazolin |  |  |  |  |  |  |  |  |  |  |  |  |  |  |  | 1 |  | 1 |  |  |  |  |  |  | 1 |  | 1 |  |  |  | 2 | 1 | 3 |  |  |  |  |  |  |
| Cefalonium |  |  |  |  | 1 | 1 |  |  |  |  |  |  |  |  |  |  |  |  |  |  |  |  |  |  |  |  |  |  |  |  | 1 |  | 1 |  |  |  |  |  |  |
| Cefuroxime |  |  |  | 1 |  | 1 | 1 |  | 1 |  |  |  |  |  |  | 1 |  | 1 |  |  |  |  |  |  | 4 |  | 4 | 3 |  | 3 | 31 | 10 | 41 |  |  |  |  |  |  |
| **IONOPHORES** |  |  |  |  | **1** | **1** |  |  |  |  |  |  |  |  |  |  |  |  |  |  |  |  |  |  |  |  |  |  |  |  |  |  |  |  |  |  |  |  |  |
| Lasalocid |  |  |  |  |  |  |  |  |  |  |  |  |  |  |  |  |  |  |  |  |  |  |  |  |  |  |  |  |  |  |  |  |  |  |  |  |  |  |  |
| Maduramycin |  |  |  |  |  |  |  |  |  |  |  |  |  |  |  |  |  |  |  |  |  |  |  |  |  |  |  |  |  |  |  |  |  |  |  |  |  |  |  |
| Monensin |  |  |  |  |  |  |  |  |  |  |  |  |  |  |  |  |  |  |  |  |  |  |  |  |  |  |  |  |  |  |  |  |  |  |  |  |  |  |  |
| Narasin |  |  |  |  |  |  |  |  |  |  |  |  |  |  |  |  |  |  |  |  |  |  |  |  |  |  |  |  |  |  |  |  |  |  |  |  |  |  |  |
| Salinomycin |  |  |  |  | 1 | 1 |  |  |  |  |  |  |  |  |  |  |  |  |  |  |  |  |  |  |  |  |  |  |  |  |  |  |  |  |  |  |  |  |  |
| Semduramicin |  |  |  |  |  |  |  |  |  |  |  |  |  |  |  |  |  |  |  |  |  |  |  |  |  |  |  |  |  |  |  |  |  |  |  |  |  |  |  |
| **LINCOSAMIDES** |  | **1** | **1** | **1** | **8** | **9** |  | **3** | **3** |  | **2** | **2** | **7** | **8** | **15** | **1** | **2** | **3** | **5** | **6** | **11** |  | **1** | **1** | **2** | **8** | **10** |  |  |  | **14** | **6** | **20** | **0** | **0** | **0** | **3** |  | **3** |
| Pirlimycin |  |  |  | 1 |  | 1 |  |  |  |  |  |  |  |  |  |  |  |  |  |  |  |  |  |  |  |  |  |  |  |  |  |  |  |  |  |  |  |  |  |
| Lincomycin |  | 1 | 1 |  | 8 | 8 |  | 3 | 3 |  | 2 | 2 | 7 | 8 | 15 | 1 | 2 | 3 | 5 | 6 | 11 |  | 1 | 1 | 2 | 8 | 10 |  |  |  | 14 | 6 | 20 |  |  |  | 3 |  | 3 |
| **PHOSPHONIC ACID** |  |  |  |  | **1** | **1** |  | **1** | **1** |  |  |  |  | **3** | **3** |  |  |  |  | **3** | **3** |  |  |  |  | **1** | **1** |  |  |  | **1** |  | **1** |  |  |  |  | **3** | **3** |
| Phosphonic Acid |  |  |  |  |  |  |  |  |  |  |  |  |  |  |  |  |  |  |  |  |  |  |  |  |  |  |  |  |  |  |  |  |  |  |  |  |  |  |  |
| Fosfomycin |  |  |  |  | 1 | 1 |  | 1 | 1 |  |  |  |  | 3 | 3 |  |  |  |  | 3 | 3 |  |  |  |  | 1 | 1 |  |  |  | 1 |  | 1 |  |  |  |  | 3 | 3 |
| **PLEUROMUTILINS** |  |  |  | **1** |  | **1** |  |  |  |  |  |  | **2** | **4** | **6** |  |  |  |  | **4** | **4** |  |  |  |  |  |  |  |  |  |  |  |  |  |  |  |  |  |  |
| Tiamulin |  |  |  | 1 |  | 1 |  |  |  |  |  |  | 2 | 4 | 6 |  |  |  |  | 4 | 4 |  |  |  |  |  |  |  |  |  |  |  |  |  |  |  |  |  |  |
| Valnemulin |  |  |  |  |  |  |  |  |  |  |  |  |  |  |  |  |  |  |  |  |  |  |  |  |  |  |  |  |  |  |  |  |  |  |  |  |  |  |  |
| **POLYPEPTIDES** | **2** | **4** | **6** | **3** | **7** | **10** | **3** | **3** | **6** |  |  |  | **1** | **4** | **5** | **4** | **2** | **6** | **1** | **4** | **5** |  | **1** | **1** | **18** | **21** | **39** |  |  |  | **15** | **4** | **19** | **2** |  | **2** |  | **1** | **1** |
| Enramycin |  |  |  |  |  |  |  |  |  |  |  |  |  |  |  |  |  |  |  |  |  |  |  |  |  |  |  |  |  |  |  |  |  |  |  |  |  |  |  |
| Gramicidin |  |  |  |  |  |  |  |  |  |  |  |  |  |  |  |  |  |  |  |  |  |  |  |  |  | 2 | 2 |  |  |  | 1 | 1 | 2 |  |  |  |  |  |  |
| Bacitracin | 2 | 2 | 4 |  | 4 | 4 |  | 1 | 1 |  |  |  | 1 | 2 | 3 |  | 2 | 2 | 1 | 2 | 3 |  | 1 | 1 | 6 | 5 | 11 |  |  |  | 7 | 1 | 8 | 1 |  | 1 |  | 1 | 1 |
| Colistin |  | 2 | 2 |  | 2 | 2 |  | 2 | 2 |  |  |  |  | 2 | 2 |  |  |  |  | 2 | 2 |  |  |  |  | 5 | 5 |  |  |  |  |  |  |  |  |  |  |  |  |
| Polymixin |  |  |  | 3 | 1 | 4 | 3 |  | 3 |  |  |  |  |  |  | 4 |  | 4 |  |  |  |  |  |  | 12 | 9 | 21 |  |  |  | 7 | 2 | 9 | 1 |  | 1 |  |  |  |
| **QUINOLONES** |  |  |  |  |  |  |  |  |  |  |  |  |  |  |  |  |  |  |  |  |  |  |  |  |  |  |  |  |  |  | **7** | **3** | **10** |  |  |  |  |  |  |
| Flumequin |  |  |  |  |  |  |  |  |  |  |  |  |  |  |  |  |  |  |  |  |  |  |  |  |  |  |  |  |  |  | 1 |  | 1 |  |  |  |  |  |  |
| Miloxacin |  |  |  |  |  |  |  |  |  |  |  |  |  |  |  |  |  |  |  |  |  |  |  |  |  |  |  |  |  |  |  |  |  |  |  |  |  |  |  |
| Nalidixic acid |  |  |  |  |  |  |  |  |  |  |  |  |  |  |  |  |  |  |  |  |  |  |  |  |  |  |  |  |  |  | 6 | 3 | 9 |  |  |  |  |  |  |
| Oxolinic acid |  |  |  |  |  |  |  |  |  |  |  |  |  |  |  |  |  |  |  |  |  |  |  |  |  |  |  |  |  |  |  |  |  |  |  |  |  |  |  |

**Table S3 (continuation)**: Number of websites where the individual antibiotics can be purchased according to the species they are intended to, classified following the OIE list of antimicrobial agents of veterinary importance (22), in the searches in English and in Spanish

|  | **All species** | | | **Bovine** | | | **Ovine** | | | **Caprine** | | | **Pigs** | | | **Horses** | | | **Poultry** | | | **Dogs** | | | **Cats and dogs** | | | **Fish** | | | **Humans** | | | **Animals and humans** | | | **Others** | | |
| --- | --- | --- | --- | --- | --- | --- | --- | --- | --- | --- | --- | --- | --- | --- | --- | --- | --- | --- | --- | --- | --- | --- | --- | --- | --- | --- | --- | --- | --- | --- | --- | --- | --- | --- | --- | --- | --- | --- | --- |
|  | **E** | **S** | **T** | **E** | **S** | **T** | **E** | **S** | **T** | **E** | **S** | **T** | **E** | **S** | **T** | **E** | **S** | **T** | **E** | **S** | **T** | **E** | **S** | **T** | **E** | **S** | **T** | **E** | **S** | **T** | **E** | **S** | **T** | **E** | **S** | **T** | **E** | **S** | **T** |
| **IMPORTANT** | | | | | | | | | | | | | | | | | | | | | | | | | | | | | | | | | | | | | | | |
| **AMINOCOUMARIN** |  |  |  |  |  |  |  |  |  |  |  |  |  |  |  |  |  |  |  |  |  |  |  |  |  |  |  |  |  |  |  |  |  |  |  |  |  |  |  |
| Aminocoumarin |  |  |  |  |  |  |  |  |  |  |  |  |  |  |  |  |  |  |  |  |  |  |  |  |  |  |  |  |  |  |  |  |  |  |  |  |  |  |  |
| Novobiocin |  |  |  |  |  |  |  |  |  |  |  |  |  |  |  |  |  |  |  |  |  |  |  |  |  |  |  |  |  |  |  |  |  |  |  |  |  |  |  |
| **ARSENICAL** |  |  |  |  |  |  |  |  |  |  |  |  |  |  |  |  |  |  |  |  |  |  |  |  |  |  |  |  |  |  |  |  |  |  |  |  |  |  |  |
| Roxarsone |  |  |  |  |  |  |  |  |  |  |  |  |  |  |  |  |  |  |  |  |  |  |  |  |  |  |  |  |  |  |  |  |  |  |  |  |  |  |  |
| Nitarsone |  |  |  |  |  |  |  |  |  |  |  |  |  |  |  |  |  |  |  |  |  |  |  |  |  |  |  |  |  |  |  |  |  |  |  |  |  |  |  |
| **BICYCLOMYCIN** |  |  |  |  |  |  |  |  |  |  |  |  |  |  |  |  |  |  |  |  |  |  |  |  |  |  |  |  |  |  |  |  |  |  |  |  |  |  |  |
| Bicyclomycin |  |  |  |  |  |  |  |  |  |  |  |  |  |  |  |  |  |  |  |  |  |  |  |  |  |  |  |  |  |  |  |  |  |  |  |  |  |  |  |
| Bicozamycin |  |  |  |  |  |  |  |  |  |  |  |  |  |  |  |  |  |  |  |  |  |  |  |  |  |  |  |  |  |  |  |  |  |  |  |  |  |  |  |
| **FUSIDIC ACID** |  |  |  |  |  |  |  |  |  |  |  |  |  |  |  |  |  |  |  |  |  |  |  |  |  |  |  |  |  |  | **6** | **2** | **8** |  |  |  |  |  |  |
| Fusidic acid |  |  |  |  |  |  |  |  |  |  |  |  |  |  |  |  |  |  |  |  |  |  |  |  |  |  |  |  |  |  | 6 | 2 | 8 |  |  |  |  |  |  |
| **ORTHOSOMYCINS** |  |  |  |  |  |  |  |  |  |  |  |  |  |  |  |  |  |  |  |  |  |  |  |  |  |  |  |  |  |  |  |  |  |  |  |  |  |  |  |
| Avilamycin |  |  |  |  |  |  |  |  |  |  |  |  |  |  |  |  |  |  |  |  |  |  |  |  |  |  |  |  |  |  |  |  |  |  |  |  |  |  |  |
| **QUINOXALINES** |  |  |  |  |  |  |  |  |  |  |  |  |  |  |  |  |  |  |  |  |  |  |  |  |  |  |  |  |  |  |  |  |  |  |  |  |  |  |  |
| Carbadox |  |  |  |  |  |  |  |  |  |  |  |  |  |  |  |  |  |  |  |  |  |  |  |  |  |  |  |  |  |  |  |  |  |  |  |  |  |  |  |
| Olaquindox |  |  |  |  |  |  |  |  |  |  |  |  |  |  |  |  |  |  |  |  |  |  |  |  |  |  |  |  |  |  |  |  |  |  |  |  |  |  |  |
| **STREPTOGRAMINS** |  |  |  |  |  |  |  |  |  |  |  |  |  |  |  |  |  |  |  |  |  |  |  |  |  |  |  |  |  |  |  |  |  |  |  |  |  |  |  |
| Virginiamycin |  |  |  |  |  |  |  |  |  |  |  |  |  |  |  |  |  |  |  |  |  |  |  |  |  |  |  |  |  |  |  |  |  |  |  |  |  |  |  |
| **THIOSTREPTON** | **1** |  | **1** |  |  |  |  |  |  |  |  |  |  |  |  |  |  |  |  |  |  |  |  |  | **1** | **2** | **3** |  |  |  |  |  |  |  |  |  |  |  |  |
| Thiostrepton | 1 |  | 1 |  |  |  |  |  |  |  |  |  |  |  |  |  |  |  |  |  |  |  |  |  | 1 | 2 | 3 |  |  |  |  |  |  |  |  |  |  |  |  |
| Nosiheptide |  |  |  |  |  |  |  |  |  |  |  |  |  |  |  |  |  |  |  |  |  |  |  |  |  |  |  |  |  |  |  |  |  |  |  |  |  |  |  |
